# Calcium‐dependent protein kinase PpCDPK29‐mediated Ca^2+^‐ROS signal and PpHSFA2a phosphorylation regulate postharvest chilling tolerance of peach fruit

**DOI:** 10.1111/pbi.70024

**Published:** 2025-02-27

**Authors:** Liangyi Zhao, Hua Cassan‐Wang, Yaqin Zhao, Yinqiu Bao, Yuanyuan Hou, Yu Liu, Zhengguo Wu, Mondher Bouzayen, Yonghua Zheng, Peng Jin

**Affiliations:** ^1^ College of Food Science and Technology Nanjing Agricultural University Nanjing China; ^2^ Laboratoire de Recherche en Sciences Végétales (LRSV) Université de Toulouse, Centre national de la recherche scientifique (CNRS), Université Toulouse III – Paul Sabatier (UPS), Toulouse‐Institut National Polytechnique (INP) Toulouse France

**Keywords:** peach fruit, chilling injury, hot water treatment, reactive oxygen species, calcium‐dependent protein kinase, phosphorylation

## Abstract

Green and chemical‐free hot water (HW) treatment can effectively reduce the chilling injury of peach fruit; however, the mechanism of inducing chilling resistance by heat treatment is still unclear. This study found that HW treatment could activate reactive oxygen species (ROS) signalling, forming ROS‐Ca^2+^ signalling. Furthermore, we identified a peach Ca^2+^ sensor, calcium‐dependent protein kinase 29 (PpCDPK29), as a positive regulator of postharvest chilling resistance. PpCDPK29 interacted with ROS‐generating proteins (PpRBOHC/D) and antioxidant enzymes (PpSOD and PpCAT1) to jointly maintain ROS homeostasis. Meanwhile, we found that PpHSFA2a was phosphorylated by PpCDPK29 and transferred to the nucleus, which enhanced the binding ability of PpHSFA2a to the target genes. Here, PpHSFA2a activated the transcription of target genes *PpHSP18.5*, *PpHSP70*, *PpGSTU7*, *PpGSTU19*, *PpGolS1* and *PpBAM1*, acted as molecular chaperones, improved ROS scavenging and enhanced osmoregulation to alleviate postharvest chilling injury of peach fruit. In summary, HW treatment could alleviate postharvest chilling injury in peach fruit by activating the PpCDPK29‐mediated Ca^2+^‐ROS and HSF‐HSP signalling pathways, providing a novel signalling network for postharvest quality control of peach fruit.

## Introduction

Peach (*Prunus persica* (L.) Batsch) is a climacteric fruit, with a high respiratory rate and increased ethylene release at room temperature after harvest, which will quickly soften, rot and deteriorate. Cold storage can effectively delay the softening and senescence of peach fruit, prolonging the postharvest life. However, peach is a typical cold‐sensitive fruit and is susceptible to chilling injury (CI), resulting in loss of flavour, reduction of juice, browning of flesh and failure to achieve normal ripening softening, thus reducing quality and commercial value (Crisosto *et al*., 1997; Lurie and Crisosto, 2005). Heat treatment is a physical, green and safe post‐harvest pretreatment method. It is widely accepted that heat treatment can reduce chilling injury symptoms and extend the shelf life of fruits and vegetables (Jin *et al*., 2009; Lauxmann *et al*., 2014; Zhao *et al*., 2023). Heat treatment increased the expression of 24 genes related to GSH metabolism, reduced oxidative stress and thus alleviated the chilling injury of banana (Si *et al*., 2022). Heat treatment increased glucose and fructose content and alleviated chilling injury in loquat fruit (Shao *et al*., 2013). Heat treatment induces arginine metabolism and increases the content of polyamines and proline, thereby significantly reducing the occurrence of chilling injury in zucchini during cold storage (Bokhary *et al*., 2020). However, the mechanism of heat treatment‐induced chilling resistance, especially the related signalling pathways, is still unclear.

Plant cold resistance involves multiple signal transduction pathways. Calcium (Ca^2+^), reactive oxygen species (ROS) signalling and protein kinases are important components of cell signal transduction processes. Generally, ROS, including singlet oxygen (^1^O_2_), hydroxyl radical (OH•), superoxide radical ion (O_2_^.**−**^) and hydrogen peroxide (H_2_O_2_), are both signalling and potentially damaging molecules, whereas the homeostasis of ROS is balanced between the complex generation system and scavenging systems (Mittler *et al*., 2022). Activation of Ca^2+^ channels by changes in membrane fluidity induced by cold stress is the main event of cold sensing. Cold‐triggered Ca^2+^ transients are sensed and decoded by different Ca^2+^ sensors, inducing changes in gene expression and ultimately ensuring cold resistance in plants (Ranty *et al*., 2016). In particular, calcium‐dependent protein kinase (CDPK/CPK) is the most differentiated calcium sensor and relies on its special structure to directly sense and respond to Ca^2+^ signalling without calmodulin (CaM). CDPK is a class of serine/threonine protein kinases with a molecular weight of 40–90 kDa, found only in plants and protists. CDPK has four important conserved domains, including the variable N‐terminal domain (VNTD), the Ser/Thr protein kinase domain (KD), the inhibitory junction domain (JD) and the C‐terminal EF‐hand containing Ca^2+^ − binding CaM‐like domain (CaM‐LD) (Cheng *et al*., 2002; Yang *et al*., 2022).

CDPKs function in signal transduction pathways mainly by phosphorylating substrates. Numerous studies have demonstrated that CDPKs respond to various biotic and abiotic stresses, such as cold, drought, high salt, high temperature, mechanical injury and pathogenic infection, implying the potential regulation of CDPKs in plant stress tolerance (Asano *et al*., 2012a; Cheng *et al*., 2002). For instance, numerous *OsCPKs* are involved in responding to abiotic stresses (Asano *et al*., 2012b; Campo *et al*., 2014; Saijo *et al*., 2000; Wei *et al*., 2014). In addition, SlCPK28 enhanced tomato heat tolerance by phosphorylating Thr‐59 and Thr‐164 of SlAPX2 (Hu *et al*., 2021b). Cold‐induced Ca^2+^ signals could be rapidly decoded by AtCPK28 and phosphorylate downstream AtNLP7 to enhance plant cold tolerance (Ding *et al*., 2022). Evidence indicates that Ca^2+^ and ROS signalling appear to be a fine inter‐regulatory network in response to various stresses, and Ca^2+^ signalling can act upstream and downstream of ROS (Steinhorst and Kudla, 2013). Ca^2+^ can activate CDPK, leading to the activation of ROS‐generating genes *RBOH* expression, which produces ROS. These ROS induce the release of Ca^2+^ from adjacent cells, and Ca^2+^ activates CDPK again to form positive feedback (Dubiella *et al*., 2013; Steinhorst and Kudla, 2013). However, the relationship between CDPK and ROS in responding to postharvest cold stress remains elusive.

Previous research demonstrated that many heat response genes also respond to low temperature, such as heat shock transcription factors (HSF) (Pons *et al*., 2014; Wang *et al*., 2017). HSFs bind to the heat shock element (HSE) sequence ‘nGAAnnTCCn’ in the promoters of many defence genes and participate in various plant stress responses, which are considered a highly redundant and flexible gene network (Åkerfelt *et al*., 2010; Guo *et al*., 2016; Haider *et al*., 2022). For example, AtHSFA1 induced the expression of stress‐responsive genes to mediate cold acclimation in *Arabidopsis* (Olate *et al*., 2018). Overexpression of *Arabidopsis AtHSF1b* or tomato *SlHSP17.7* improves cold tolerance in transgenic tomatoes (Li *et al*., 2023; Zhang *et al*., 2020). Overexpression of *SPL7OX* encoding HSF can enhance the cold tolerance of rice (Hoang *et al*., 2019). In our previous study, HW activated the expression of *PpHSFA1b*, *PpHSFA2a*, *PpHSFA4a*, *PpHSFA4b*, *PpHSFB1a*, *PpHSFB2b* and *PpHSFC1a* to confer peach fruit cold tolerance. In particular, the expression of *PpHSFA2a* dramatically increased during cold storage (Wang *et al*., 2022). However, the specific functions of HW‐induced PpHSFA2a in peach fruit cold tolerance and whether it interacts with Ca^2+^ signals remain unclear.

Here, we have substantially resolved the missing part of the regulatory mechanism of HW‐induced peach fruit chilling resistance via CDPK‐mediated Ca^2+^‐ROS signalling. Our study has identified the previously unrecognized function of PpCDPK29 that promoted ROS production and scavenging during postharvest cold response signalling. Furthermore, PpCDPK29 could phosphorylate PpHSFA2a and enhance its regulation of downstream defence genes to alleviate peach fruit postharvest chilling injury. In summary, this study established a regulatory module of HW‐induced chilling resistance mechanisms, including the activation of Ca^2+^‐ROS, PpCDPK29, and its substrate PpHSFA2a to alleviate peach fruit postharvest chilling injury.

## Results

### Hot water treatment induces ROS–Ca^2+^ signalling to enhance cold tolerance of peach fruit

**Figure 1:**
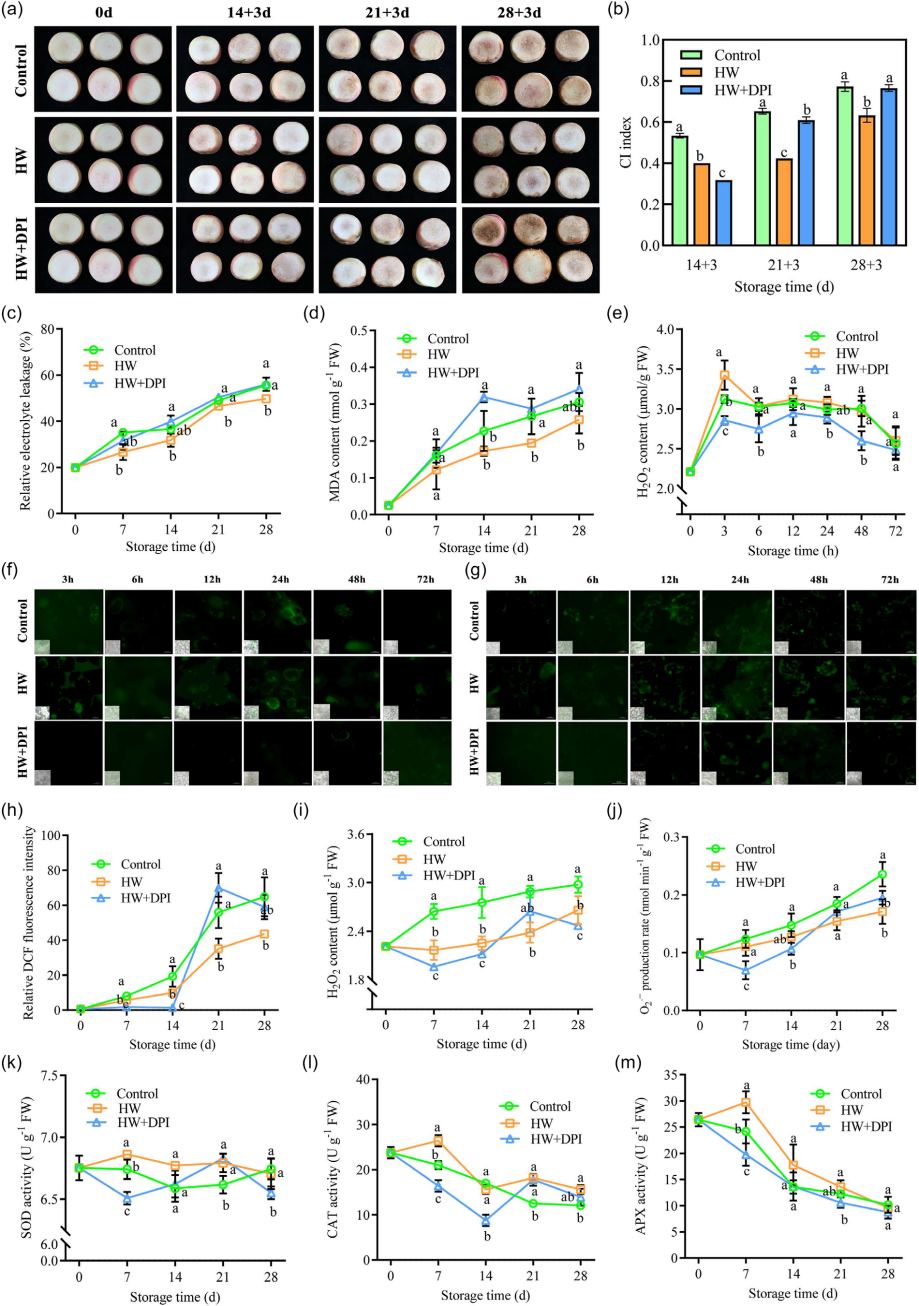
Effect of HW and HW + DPI treatment on peach fruit during cold storage. (a, b) The internal appearance (a) and chilling injury CI index (b) of peach fruit after the 3 days shelf life after cold storage. (c, d) Relative electrical conductivity (c) and MDA content (d) in cold‐store peach fruit. (e) H_2_O_2_ content of peach fruit in the early cold storage stage. (f, g) The H_2_DCF‐DA fluorescent probe detected ROS (f) and Fluo‐3/AM detected intracellular Ca^2+^ (g) of peach fruit in the early cold storage stage (Bars: 50 mm). (h–j) ROS relative fluorescence intensity (h), H_2_O_2_ content (i), O_2_^.**−**^ production rate (j) during cold‐store peach fruit. (k‐m) SOD (k), APX (l) and CAT (m) activities during cold‐stored peach fruit. Dates are presented as means ± SD (*n* = 3). Different lowercase letters indicate significant differences between groups (*P* < 0.05).

To investigate the function of ROS in HW‐induced peach fruit cold tolerance, ‘Hujingmilu’ peach was treated with HW and HW combined with Diphenyleneiodonium chloride (DPI, a specific inhibitor of NADPH oxidase, commonly used to inhibit ROS production). After 14 days of cold storage, peach fruit began to brown slightly, and the internal browning gradually aggravated as time progressed. Compared with the control group, HW treatment significantly inhibited the browning of peach fruit flesh, while HW + DPI treatment aggravated the browning of peach fruit flesh at the later stage (Figure 1a). Consistent with visual inspection, HW treatment significantly inhibited the browning of peach fruit flesh throughout the storage, while HW + DPI treatment inhibited the browning of peach fruit flesh at the early stage until 21 days but aggravated at the later stage at 28 days (Figure 1b–d). To understand whether ROS production in cold‐stored peaches was affected by HW treatment, H_2_O_2_ content and ROS levels were measured. HW triggered a rapid H_2_O_2_ accumulation in peaches at 3 h of cold storage, while a reduction was observed in the HW + DPI group throughout the early stage (Figure 1e). ROS accumulation detected by DCF relative fluorescence intensity showed similar tendencies in HW treatment that peaked at 3 and 24 h of cold storage (Figures 1f and S1a). Moreover, cold storage also triggered an increase in cytoplasmic Ca^2+^ concentration in peaches, which peaked at 24 h (control group), and a similar increase with a greater amplitude was found with HW treatment, while HW + DPI caused a reduction in cytoplasmic Ca^2+^ levels, probably by inhibiting ROS signalling (Figures 1g and S1b).

In addition, the ROS of the HW treatment group was lower than that of the control group throughout the storage period. While the HW + DPI treatment led to a decrease in ROS levels at 7 days, it gave rise to a sudden increase at a later stage (Figures 1h, j and S1c). The result suggested that the HW + DPI treatment decreased the ROS production during the early stage but triggered an accelerated production of ROS in the later stage (14–28 days). We further examined the ROS scavenging systems during storage; HW treatment increased the activities of ROS scavenging enzymes superoxide dismutase (SOD), catalase (CAT) and ascorbate peroxidase (APX) throughout the storage (Figure 1k–m). We observed that in the early stage, HW + DPI treatment decreased the activities of these antioxidant enzymes as well as the ROS levels. The accelerated ROS production at a later stage (around 21 days) was accompanied by a sudden increase in SOD and CAT activities. Taking together the results of the highest endoplasmic Ca^2+^ concentration in peach during storage after HW treatment (Figures 1g and S1d,e), we therefore speculate that HW triggered activated Ca^2+^ signalling and higher ROS levels, which stimulate the ROS scavenging system more efficiently, thereby leading to enhanced cold tolerance of peaches during storage after HW treatment.

### PpCDPK29 can positively regulate cold resistance

**Figure 2:**
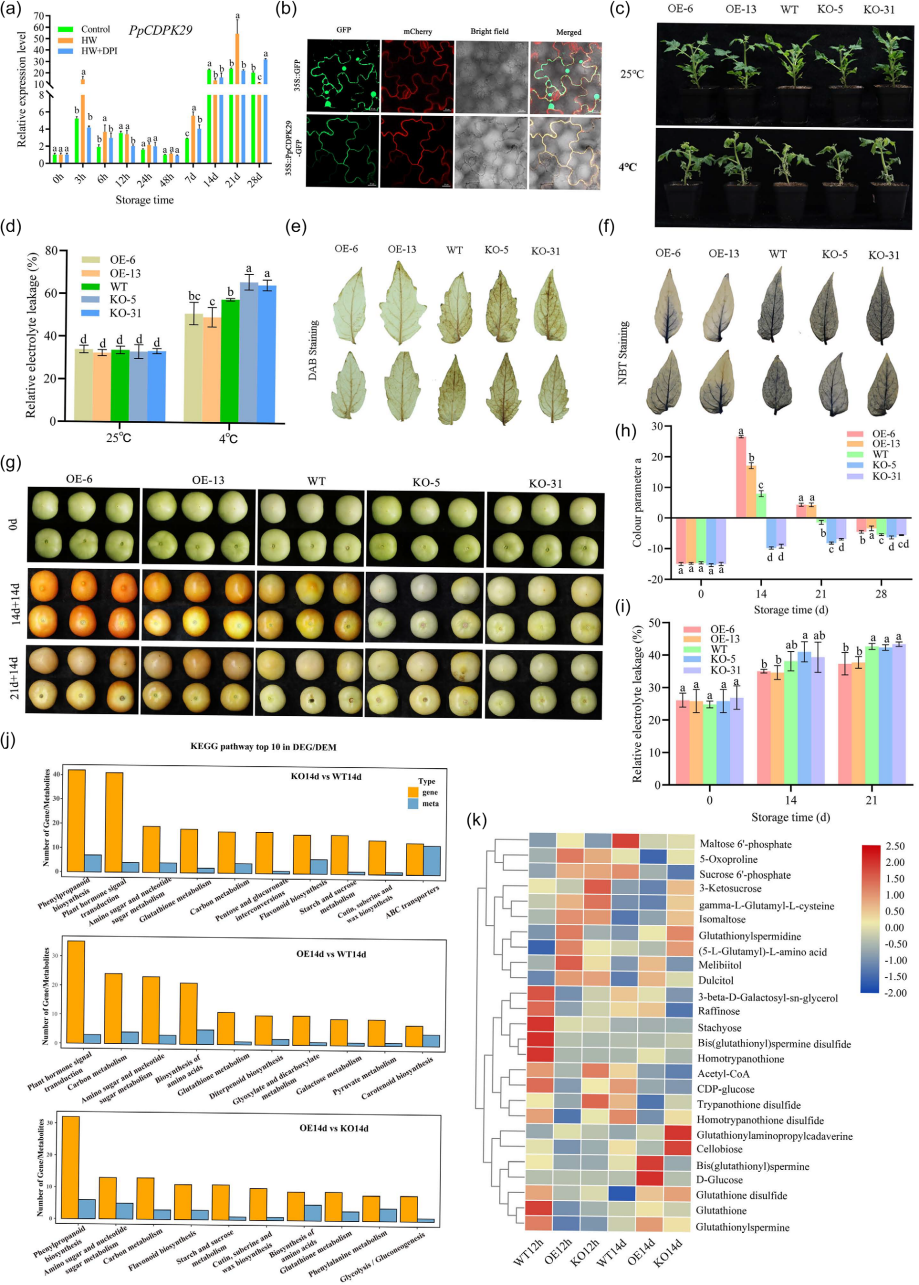
Analysis of cold resistance of transgenic tomatoes. (a) Relative expression pattern of PpCDPK29 of peach fruit during cold storage. (b) Subcellular localization of PpCDPK29 (Bars: 20 μm). (c‐d) The phenotype of low‐temperature treatment (c) and relative electrolyte leakage (d) of WT and transgenic tomato lines. (e, f) DAB (e) and NBT (f) histochemical staining in tomato leaves after low‐temperature stress. (g) Chilling injury symptoms in WT and transgenic tomato fruit after cold treatment. (h, i) Colour parameter a (h) and relative electrolyte leakage (i) of WT and transgenic tomato fruit after cold treatment. (j) KEGG pathway top 10 in DEG/DEM of WT and transgenic tomato fruits after 14 days of low temperature, and comparison groups were KO14 versus WT14, OE14 versus WT14 and OE14 versus KO14. (k) Heat map of DEMs in Glutathione metabolism, Starch and sucrose metabolism and Galactose metabolism pathway. The colour parameter a measure the intensity of red or green substances, a > 0 means the colour is red, and a < 0 means the colour is green. Different lowercase letters indicate significant differences between groups (*P* < 0.05).

We further investigate if and how ROS–Ca^2+^ signalling is activated to enhance cold tolerance after HW treatment. As major members of Ca^2+^ sensors, 17 CDPKs in the peach genome have been identified in previous studies. PpCDPK29, formerly also known as PpCDPK2, was identified to be highly responsive to both HW and CaCl_2_ treatment (Zhao *et al*., 2022), thus it was selected for further study. PpCDPK29 has the highest homology with CDPK29 in *Arabidopsis* and tomato, so it is now referred to as PpCDPK29. Protein sequence analysis and multiple sequence alignment showed that PpCDPK29 contained a highly conserved protein kinase domain and four EF‐hand Ca^2+^‐binding motifs, corresponding to a canonical member of the *CDPK* gene family (Figure S2a,b). *PpCDPK29* was rapidly and transiently induced by the low temperature of cold storage and even more strongly induced by HW treatment + cold storage (Figure 2a). Protein subcellular localization assay showed that PpCDPK29 was targeted exclusively to the cell membrane (Figure 2b), which is consistent with its supposed function as a signal sensor. We further characterized the PpCDPK29 function using reverse genetic approaches. As no stable genetic transformation has been established in peaches, we took advantage of the efficient stable transformation system of tomato (Micro‐Tom), which is also the model system for fleshy fruit research. We thereby overexpressed the peach gene *PpCDPK29* in tomato and used the CRISPR/Cas9 technique to generate *CDPK29*‐knockout (KO) lines targeting its homologous gene *SlCDPK29* in tomato. The following experiments used two overexpression (OE) lines of tomato (OE‐6 and OE‐13) showing the highest overexpression level of peach gene *PpCDPK29* and two homozygous SlCDPK29‐KO mutant lines CDPK29‐5 and CDPK29‐31 (KO‐5 and KO‐31) (Figure S3a,b). Whole plants of OE and KO tomato lines were subjected to a low temperature treatment at 4 °C for 14 days. Visually, the wild type (WT) and KO lines had a more serious chilling injury and most of their leaves were damaged, while the OE lines had lighter chilling injury symptoms after cold stress (Figure 2c). Consistently, the relative electrolyte leakage of the KO lines was the highest (66.06% and 64.62%), while that of the OE lines was the lowest (51.05% and 49.46%) (Figure 2d). Moreover, 3,3'‐diaminobenzidine (DAB) and nitroblue tetrazolium (NBT) histochemical staining were used to detect the contents of H_2_O_2_ and O_2_^.**−**^, respectively. The DAB and NBT staining in the leaves of OE tomato lines was lighter than that of WT and KO lines (Figure 2e,f), indicating that the OE lines accumulated less H_2_O_2_ and O_2_^.**−**^, while the KO lines had the deepest staining and the most serious oxidative stress.

We further investigated whether PpCDPK29 regulates cold tolerance in postharvest fruits. Mature green tomato fruits were subjected to low‐temperature stress at 4 °C. The chilling injury of tomatoes prevents the fruit from ripening normally, with damaging brown spots appearing on the surface of the fruit. The fruits of KO lines showed serious chilling injury symptoms, and none of the fruits turned red after 14 and 21 days of low‐temperature treatment and 14 days of storage at room temperature (Figure 2g); colour parameter a remained negative, further validating our visual observation (Figure 2h). WT fruit ripening was also impaired, showing green fruit with brown spots appearing after 21 days of cold stress and 14 days of storage at room temperature. As expected, the fruits of OE lines exhibiting less chilling injury symptoms turned red or orange‐yellow after 14 and 21 days of low‐temperature treatment and 14 days of storage at room temperature (Figure 2g,h). Besides, the relative electrolyte leakage of OE fruits was significantly lower than that of WT and KO (Figure 2i). These results collectively suggested that the overexpression of the PpCDPK29 enhanced cold resistance in tomato fruits.

To further explore the molecular mechanism of CDPK29 positive regulation of cold tolerance, tomato fruit stored at low temperature (4 °C) for 12 h and 14 days was used for transcriptome analysis. Principal component analysis (PCA) clearly distinguishes the samples from different treatments as well as groups the biological repetitions together, showing high sample repeatability (Figure S4a). A total of 7417 differentially expressed genes (DEGs) were found in the six comparison groups (Figure S4b). Venn analysis showed that there were 84 and 106 DEGs in the three comparison groups after low‐temperature storage for 12 h and 14 days, respectively (Figure S4c,d). The Gene Ontology (GO) and Kyoto Encyclopedia of Genes and Genomes (KEGG) analysis found that DEGs were mainly enriched in secondary metabolic process, galactose metabolism, glutathione metabolism, MAPK signalling pathway‐plant and other pathways (Figure S5). To identify the metabolites associated with cold tolerance, we performed non‐targeting metabolomics analysis on tomato fruits at 12 h and 14 days after low temperature storage. The Spearman rank correlation coefficient (r) of the samples in the group was close to 1, indicating that the differently expressed metabolites (DEMs) were relatively reliable (Figure S5a). A total of 4183 metabolites were detected in the metabolome, and KO14d versus WT14d had the most DEMs with 964 (Figure S5b). KEGG analysis revealed that DEMs were mainly enriched in various biosynthesis of various plant secondary meta, arginine and proline meta, amino sugar and nucleotide sugar meta (Figure S5c).

The transcriptome and metabolome cross‐analysis was performed on 14 days samples. There were 69, 52 and 62 metabolic pathways in the transcriptome and metabolome in KO14d versus WT14d, OE14d versus WT14d and OE14d versus KO14d, respectively (Figure S7a). The top 10 KEGG of DEGs and DEMs in the comparison group included plant hormone signal transduction, phenylpropanoid biosynthesis, glutathione metabolism, carbon metabolism, flavonoid biosynthesis, starch and sucrose metabolism and galactose metabolism (Figure 2j). ROS metabolism and sugar metabolism play important roles in chilling injury; we therefore further examined DEGs and DEMs in ROS metabolism‐related glutathione metabolism and sugar metabolism‐associated starch & sucrose metabolism and galactose metabolism. Heatmap analysis showed that glutathione S‐transferase and beta‐galactosidase genes, and small molecular weight sugars such as raffinose, D‐glucose, melibiitol, dulcitol and glutathione content were significantly upregulated in OE14d (Figures 2k and S7b). The results indicate that glutathione metabolism and galactose metabolism may play an important role in PpCDPK29 alleviating chilling injury in tomatoes.

### PpCDPK29 is involved in ROS production and scavenging through the Ca^2+^–ROS signalling pathway

**Figure 3:**
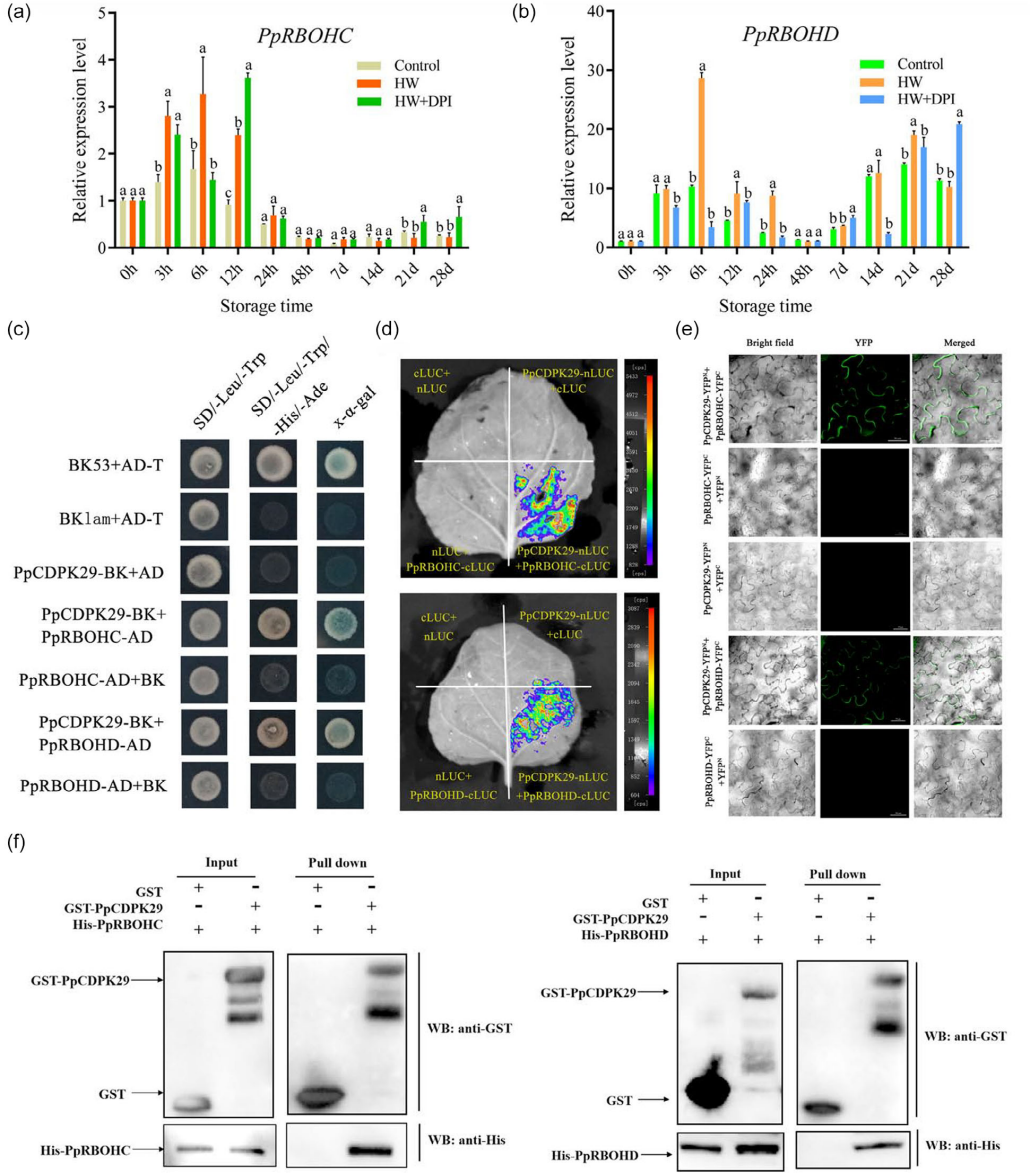
Interaction verification of PpCDPK29 and PpRBOHC/D. (a‐b) Gene expression analysis of PpRBOHC and PpRBOHD. (c–f) Interaction verification between PpCDPK29 and PpRBOHC/D by yeast two‐hybrid (c), LCI (d), BiFC (Bars: 50 μm) (e) and pull‐down (f) assays. Different lowercase letters indicate significant differences between groups (*P* < 0.05).

To further elucidate the regulatory complex of PpCDPK29, we performed a yeast two‐hybrid (Y2H) screen with the Variable N‐terminal domain (VD) and the kinase domain (KD) of PpCDPK29‐1 as bait (Figure S8). Notably, we identified four ROS‐associated proteins, PpRBOHD, PpRBOHC, PpSOD and PpCAT1 among the positive clones. Firstly, we performed quantitative real‐time PCR (qRT‐PCR) analysis to verify the expression of these genes. The expression level of two genes, *PpRBOHD* and *PpRBOHC*, was rapidly and transiently induced by HW treatment (Figure 3a,b). Next, the targeted Y2H assay confirmed interactions among PpCDPK29 and PpRBOHD/C. Only the positive control and cotransformants (PpCDPK29‐BK and PpRBOHD/C‐AD) grew on SD/‐Ade/‐His/‐Leu/‐Trp medium and showed blue coloration after X‐*α*‐gal staining (Figure 3c). Moreover, luciferase complementation imaging (LCI) found that stronger luciferase fluorescence was emitted in PpCDPK29‐nLUC and PpRBOHD/C‐cLUC cotransformed tobacco leaves, whereas no fluorescence was observed in the controls (Figure 3d). Similarly, bimolecular fluorescence complementation (BiFC) assays found that YFP fluorescence was only observed on the cell membrane of tobacco leaves infected by PpCDPK29‐YFP^N^ and PpRBOHD/C‐YFP^C^ (Figure 3e). Finally, the interaction was further validated by the GST pull‐down assay. The empty GST protein could not bind to the His‐PpRBOHD/C protein and the GST‐PpCDPK29 and His‐PpRBOHD/C proteins could bind in vitro (Figure 3f). These results demonstrate the interaction of PpCDPK29 with PpRBOHD and PpRBOHC proteins in vitro and in vivo to promote ROS production.

**Figure 4:**
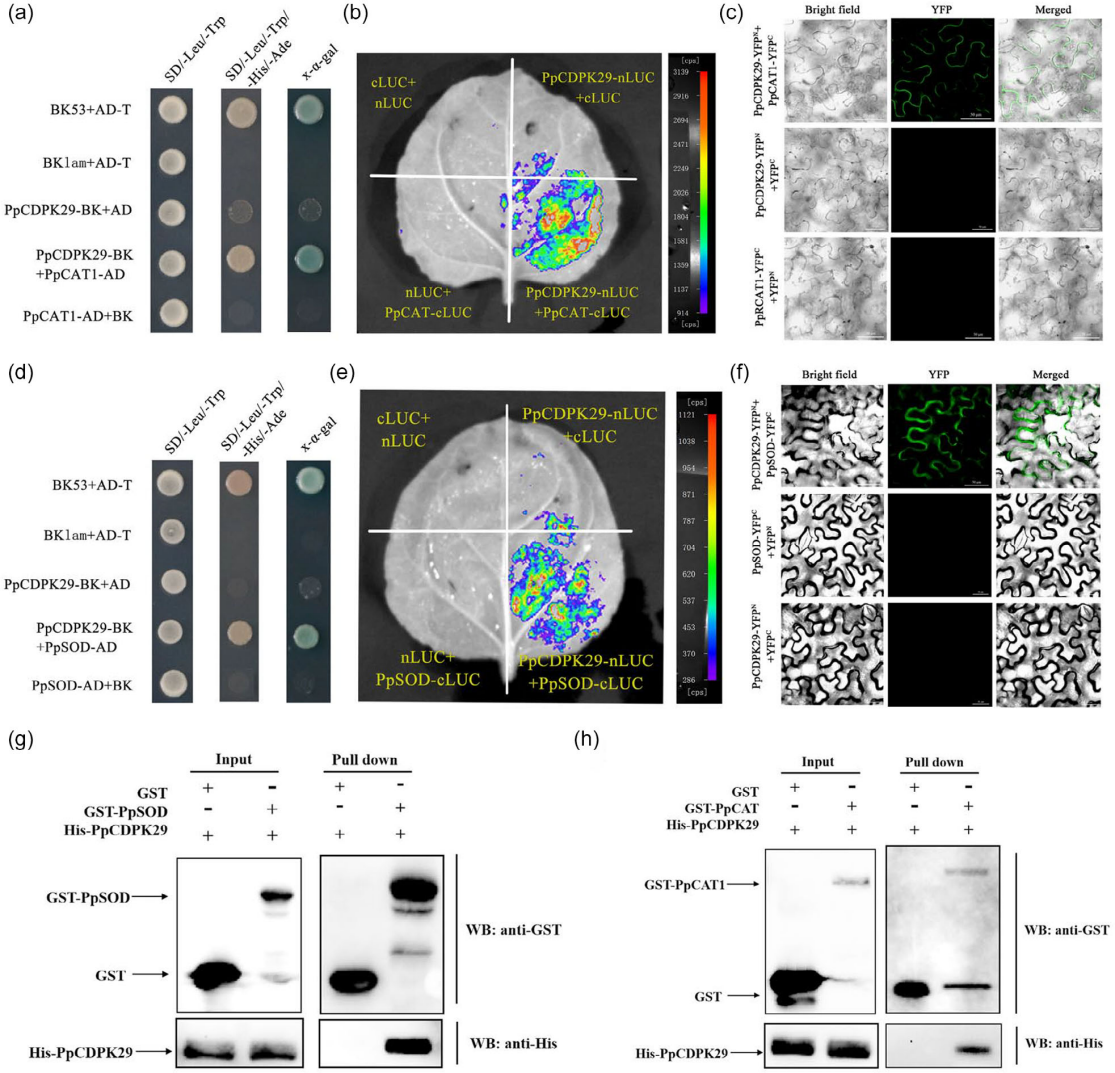
PpCDPK29 interacted with PpCAT1 and PpSOD. (a–c) Yeast two‐hybrid (a), LCI (b) and BiFC (Bars: 50 μm) (c) assay verified the interaction between PpCDPK29 and PpCAT1. (d, f) Yeast two‐hybrid (d), LCI (e) and BiFC (Bars: 50 μm) (f) assay verified the interaction between PpCDPK29 and PpSOD. (g, h) Pull‐down assay verifies that PpCDPK29 interacted with PpCAT1 (g) and PpSOD (h).

Furthermore, HW treatment also activated the expression of *PpCAT1* and *PpSOD* (Figure S9a,b). Y2H assay showed that yeast co‐transfected with PpCDPK29‐BK and PpCAT1‐AD plasmids grew on the selective medium, indicating that PpCDPK29 and PpCAT1 could interact in yeast (Figure 4a). YFP fluorescence was observed in the cells of tobacco leaves co‐infected with pCDPK29‐YFP^N^ and PpCAT1‐YFP^C^ (Figure 4b), and stronger fluorescence was observed only in tobacco leaves co‐infected with PpCDPK29‐nLUC and PpCAT1‐cLUC (Figure 4c), suggesting that PpCDPK29 and PpCAT1 could interact in plants. Likewise, the Y2H, BiFC and LCI assays showed that PpCDPK29 can interact with PpSOD (Figure 4d–f). Consistently, the GST pull‐down assay showed that the GST‐PpCDPK29 protein could interact with His‐PpCAT1 and His‐PpSOD fusion proteins (Figure 4g,h). In summary, Y2H, LCI, BiFC and GST pull‐down assays demonstrated that PpCDPK29 interacted with PpSOD and PpCAT1 to enhance antioxidant capacity and scavenge excessive ROS.

### PpCDPK29 phosphorylates PpHSFA2a

**Figure 5:**
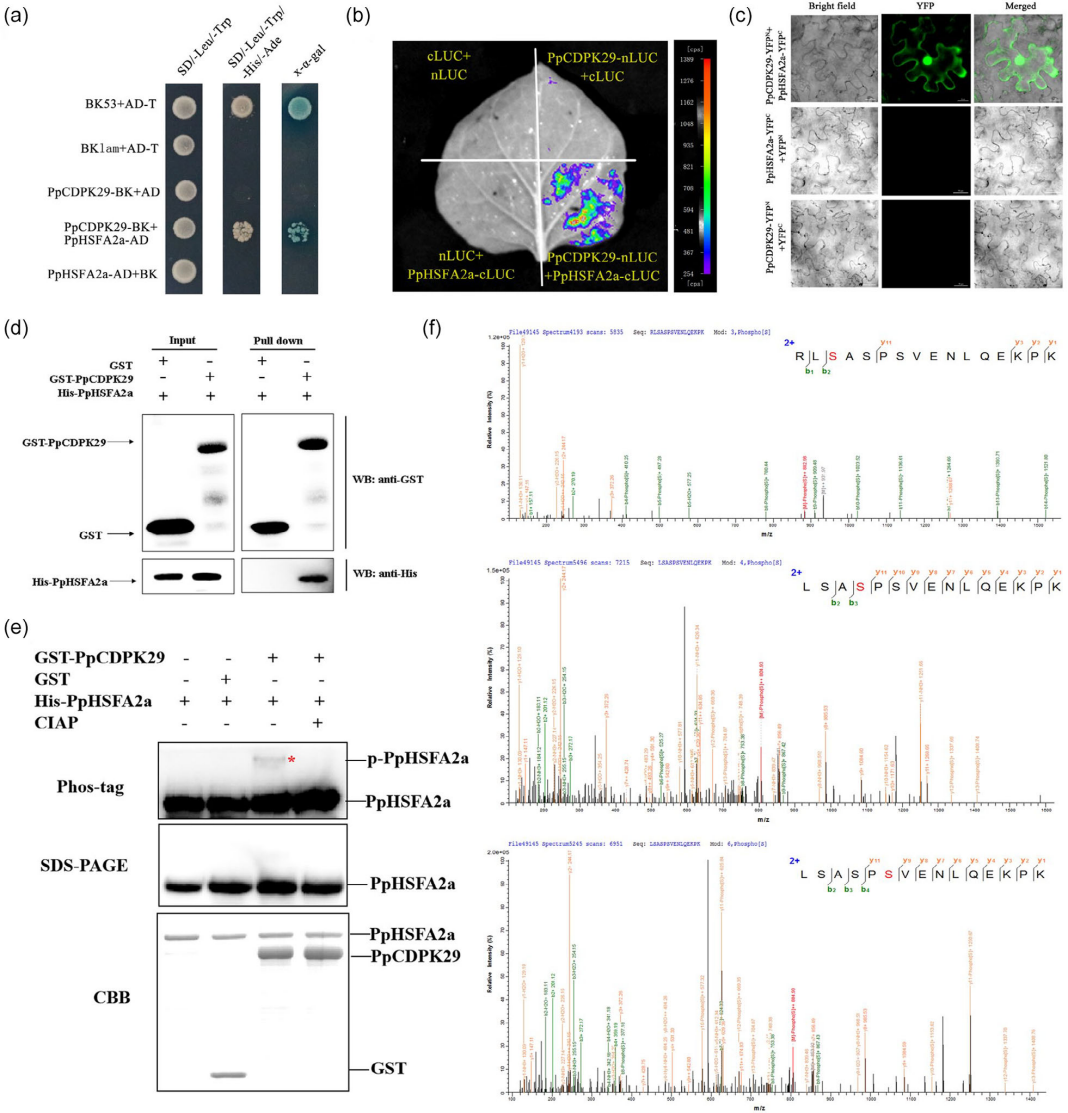
PpCDPK29 phosphorylates PpHSFA2a. (a–d) Yeast two‐hybrid (a), LCI (b), BiFC (Bars: 50 μm) (c) and pull‐down (d) assays verified the interaction between PpCDPK29 and PpHSFA2a. (e) In vitro transphosphorylation between PpCDPK29 and PpHSFA2a. Phos‐tag refers to the WB of PAGE containing Phos‐tag, SDS‐PAGE is the WB of ordinary gel, and CBB is the Coomassie Brilliant Blue staining result of the protein. (f) Mass spectrometry analysis of PpHSFA2a phosphorylation sites. Phos‐tag is a bicyclic metal complex that can delay the migration of phosphorylated proteins in SDS‐polyacrylamide gel electrophoresis (PAGE), thereby distinguishing phosphorylated and non‐phosphorylated proteins. The * showed phosphorylated PpHSFA2a.

Interestingly, PpHSFA2a was also an interaction candidate for PpCDPK29. PpHSFA2a has the closest evolutionary relationship with AtHSFA2 in *Arabidopsis thaliana*. PpHSFA2a belongs to class A HSF, including five basic functional domains: DNA binding domain (DBD), oligomerization domain (OD) or HR‐A/B, nuclear localization signal domain (NLS), nuclear export signal (NES) and activator peptide motif (AHA) (Figure S10a). HW treatment could significantly activate the expression of PpHSFA2a, especially at 3 h of storage, and PpHSFA2a was also induced by low temperature (Figure S10b). Firstly, the Y2H assay result revealed that PpCDPK29‐BK and PpHSFA2a‐AD co‐transformed yeast could grow on the selective medium and stain blue by X‐*α*‐gal (Figure 5a). The LCI assay showed that the co‐transformation of PpCDPK29‐nLUC and PpHSFA2a‐cLUC observed fluorescence in tobacco leaves (Figure 5b). In addition, the exogenous addition of CaCl_2_ enhanced the fluorescence intensity of the co‐transformation of PpCDPK29 and PpHSFA2a. In contrast, adding EGTA weakened the fluorescence intensity (Figure S11), indicating that the interaction between PpCDPK29 and PpHSFA2a was Ca^2+^ dependent. The BiFC assay showed that co‐transformed pCDPK29‐YFP^N^ and PpHSFA2a‐YFP^C^ emitted YFP fluorescence in the cytoplasm and nucleus, whereas no YFP fluorescence was detected for other negative controls (Figure 5c). We confirmed this result with the GST pull‐down assay. Purified PpHSFA2a protein with His‐tag could be pulled down by GST‐tagged PpCDPK29 protein, but could not be pulled down by the GST‐tagged empty protein, indicating that PpCDPK29 could interact with PpHSFA2a (Figure 5d).

PpCDPK29 interacted with PpHSFA2a, and PpHSFA2a might be a substrate for phosphorylation by PpCDPK29. Accordingly, the purified GST‐PpCDPK29 and His‐PpHSFA2a proteins were phosphorylated in vitro. Phos‐tag SDS‐PAGE showed no phosphorylation band in His‐PpHSFA2a and the empty GST reaction. A shifted band was observed when His‐PpHSFA2a was incubated with activated GST‐PpCDPK29. After adding calf intestinal alkaline phosphatase (CIAP), the band of His‐PpHSFA2a with slower migration disappeared, indicating that this migration was the result of phosphorylation (Figure 5e). The phosphorylation assay showed that PpCDPK29 could phosphorylate PpHSFA2a in vitro. Moreover, the possible phosphorylation sites of PpHSFA2a were identified by liquid chromatography–tandem mass spectrometry (LC–MS/MS) analysis. PpHSFA2a identified three serine (Ser243, Ser245 and Ser247) sites that PpCDPK29 could phosphorylate in vitro, and all three phosphorylation sites are located in the nuclear localization (NLS) functional domain (EIGRKRRLSASPS peptide) (Figure 5f). However, the effect of phosphorylation on the function of PpHSFA2a needs further exploration.

### Characteristic analysis of PpHSFA2a

**Figure 6:**
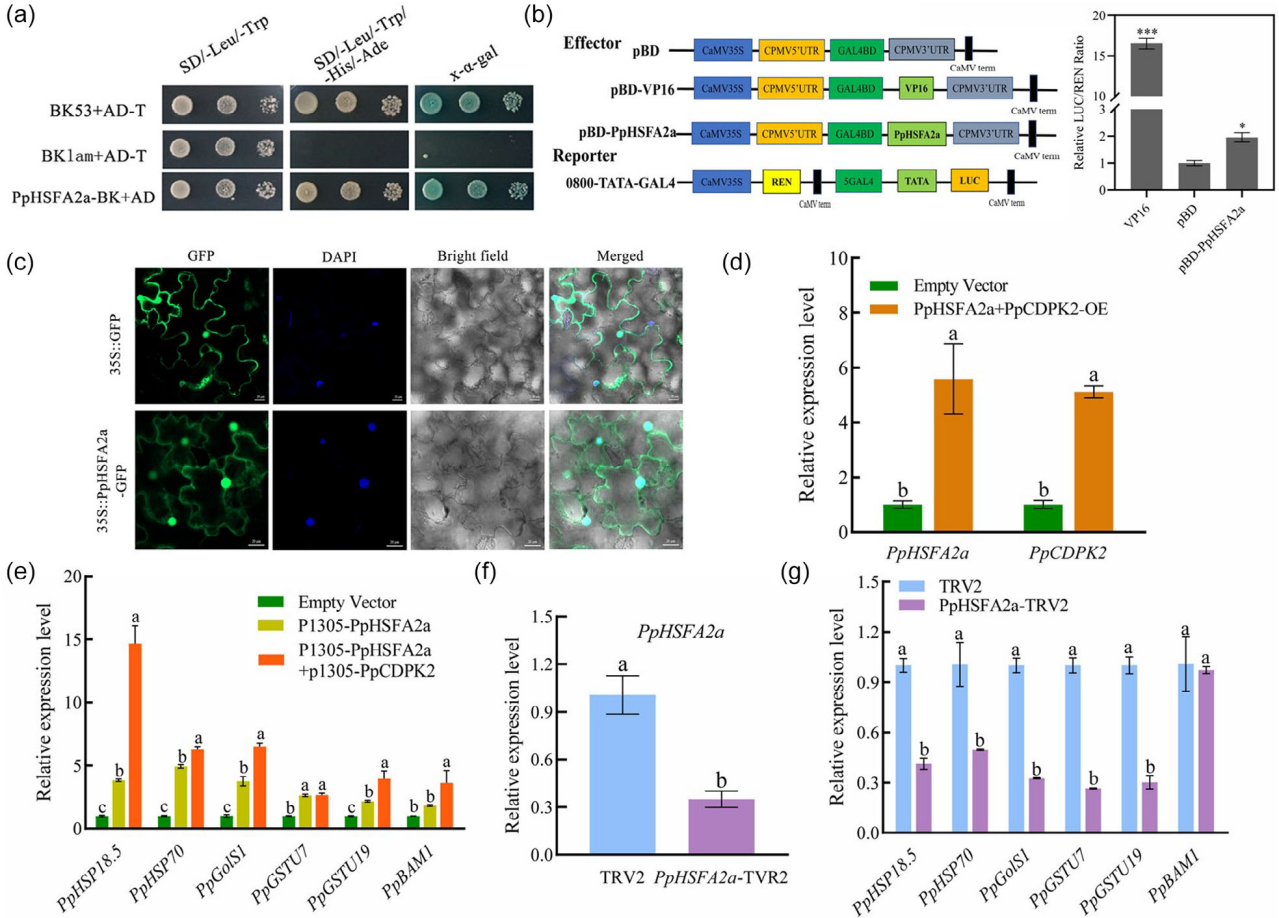
Protein characterization of PpHSFA2a. (a) Validation of self‐activation of PpHSFA2a in yeast. (b) Transcription activity of PpHSFA2a. (c) Subcellular localization of PpHSFA2a (Bars: 20 μm). (d) The expression levels of *PpHSFA2a* and *PpCDPK29* after co‐overexpression PpHSFA2a and PpCDPK29. (e) The expression levels of *PpHSP18.5*, *PpHSP70*, *PpGSTU7*, *PpGSTU19*, *PpGolS1* and *PpBAM1* genes after transient overexpression of *PpHSFA2a* and co‐overexpression of *PpHSFA2a* and *PpCDPK29*. (f) The expression levels of *PpHSFA2a* after silencing PpHSFA2a by VIGS. (g) The expression levels of *PpHSP18.5*, *PpHSP70*, *PpGSTU7*, *PpGSTU19*, *PpGolS1* and *PpBAM1* genes after transient silencing of *PpHSFA2a*. The * and *** indicate significant differences at *P* < 0.05 or *P* < 0.001. Different lowercase letters indicate significantly different values (*P* < 0.05).

We studied the transcriptional activity of PpHSFA2a. The yeast strains transfected with PpHSFA2a‐BK and AD could grow normally on SD/‐Ade/‐Leu/‐Trp/‐Ura medium. Furthermore, the LUC/REN ratio of the pBD‐VP16 and pBD‐PpHSFA2a groups was significantly higher than that of the pBD‐pEAQ empty, indicating that PpHSFA2a had self‐activation and transcriptional activation activity (Figure 6a,b). As expected, PpHSFA2a had transcriptional activation activity and had the characteristics of class A HSF subfamily. Next, we investigated the subcellular localization of PpHSFA2a. The GFP fluorescence of PpHSFA2a‐GFP was observed in the cytoplasm and nucleus (Figure 6c). As a transcription factor, HSFs generally play a role in the nucleus. We investigated whether temperature stress causes changes in the localization of PpHSFA2a. Intriguingly, we observed strong fluorescence signals of PpHSFA2a‐GFP mainly in the nucleus under high‐temperature conditions (37 °C). Under low‐temperature (4 °C) conditions, the localization change of PpHSFA2a‐GFP was not obvious, and only a small part was transferred to the nucleus (Figure S12). The results indicate that high temperature can cause changes in the localization and induce nuclear translocation of PpHSFA2a.

To investigate the role of PpHSFA2a in regulating *HSPs* and other stress‐related genes, according to the transcriptome analysis after HW treatment in the previous article (Zhao *et al*., 2023) and combined transcriptome and metabolome analysis (Figure 2k and S7b), the DEGs (*PpHSP18.5, PpHSP70, PpGSTU7, PpGSTU19, PpGolS1* and *PpBAM1*) with promoter‐containing HSE‐binding elements in HSPs, sugar and glutathione metabolism were screened. HW treatment significantly activated the expression of *PpHSP18.5* and *PpHSP70* genes, and the expression trend was consistent with *PpHSFA2a* (Figure S13a,b). HW treatment significantly activated the up‐regulation of *PpGSTU7* and *PpGSTU19* at the late stage (Figure S13c,d). The expression of the *PpGolS1* gene was up‐regulated by HW treatment, which was 4.7‐fold higher than the control at 21 days (Figure S13e). In addition, both cold storage and HW treatment activated the expression of *PpBAM1* (Figure S13f). Next, transient overexpression and silencing of the *PpHSFA2a* gene in peach fruit and analysis of the expression of downstream genes were conducted. Green fluorescence could be observed in peach fruit after transient overexpression of the GFP empty vector and *PpHSFA2a* gene, indicating that transient infection of the *PpHSFA2a* could correctly express the protein in peach fruit (Figure S14a,b). After transient overexpression of PpHSFA2a, the expression level of the *PpHSFA2a* gene was the highest after 2 days of room temperature and 14 days of low temperature storage (Figure S14c). The expression levels of downstream‐related genes were analysed using this sample. The expression levels of *PpHSFA2a* and *PpCDPK29* were significantly increased after co‐infection with PpCDPK29 and PpHSFA2a (Figure 6d). The expression levels of *PpHSP18.5*, *PpHSP70*, *PpGSTU7*, *PpGSTU19*, *PpGolS1* and *PpBAM1* genes were also significantly increased by overexpression of *PpHSFA2a*, and further improved by co‐infection with PpCDPK29 (Figure 6e). In contrast, the expression of *PpHSFA2a*, *PpHSP18.5*, *PpHSP70*, *PpGSTU7*, *PpGSTU19* and *PpGolS1* genes was significantly decreased after VIGS‐mediated silencing (Figure 6f,g). The results indicate that these genes may be directly regulated by PpHSFA2a.

### Phosphorylation enhances PpHSFA2a binding to downstream genes

**Figure 7:**
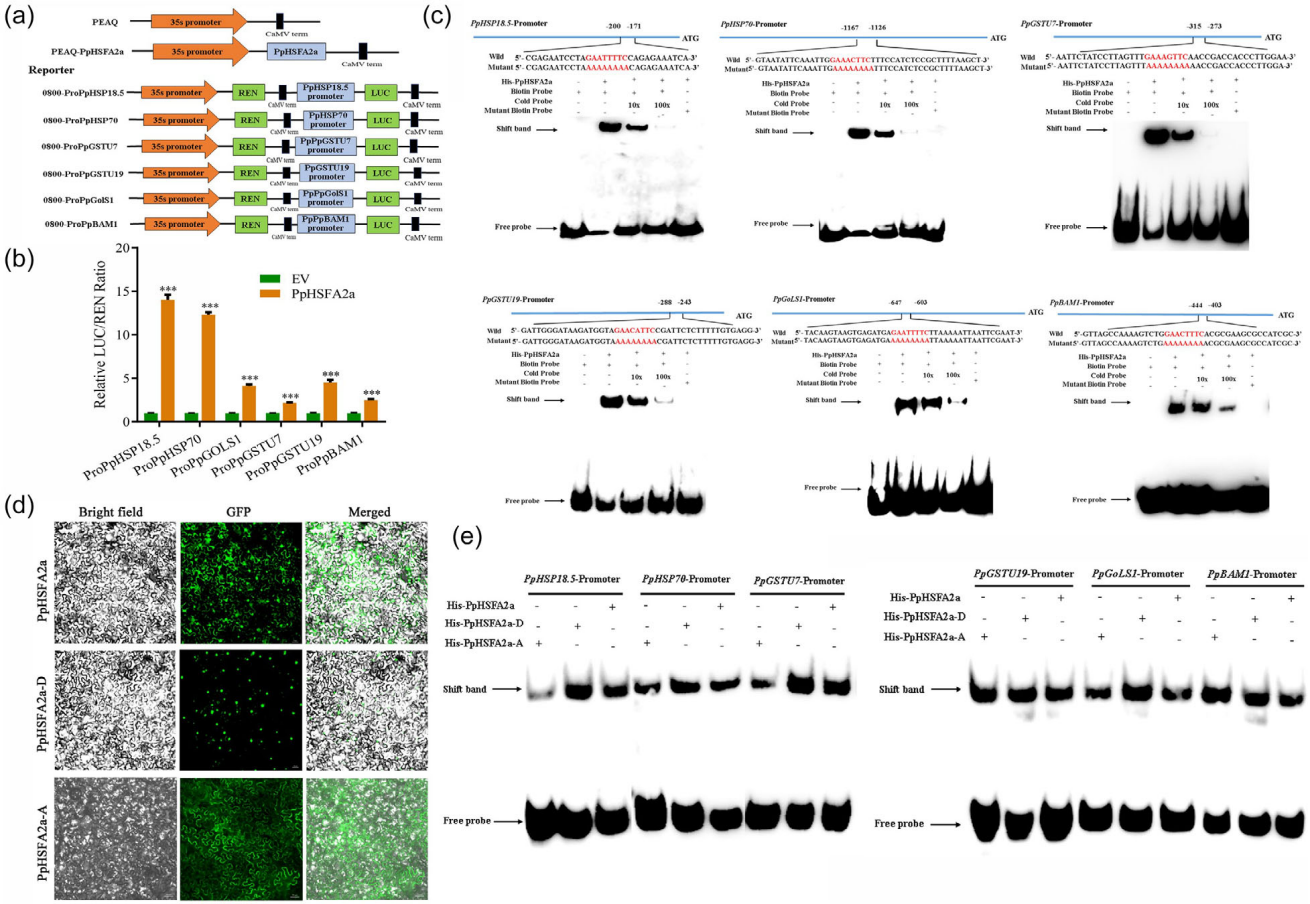
PpHSFA2a regulated downstream defence genes by binding to the HSE elements. (a) Schematic diagram of the effector and two reporter constructs used for DLR. (b) DLR verified the transcriptional activation of PpHSFA2a on *PpHSP18.5*, *PpHSP70*, *PpGSTU7*, *PpGSTU19*, *PpGoSL1* and *PpBAM1*. (c) EMSA analysis showed the PpHSFA2a bound to the HSE elements of *PpHSP18.5*, *PpHSP70*, *PpGSTU7*, *PpGSTU19*, *PpGolS1* and *PpBAM1* promoters. (d) Phosphorylation‐mediated plasma nucleus migration of PpHSFA2a. The Ser of the three phosphorylated sites of PpHSFA2a was mutated into the non‐phosphorylated alanine (Ala), PpHSFA2a‐A, to mimic the non‐phosphorylated state. Meanwhile, the Ser of the three phosphorylation sites of PpHSFA2a was mutated to aspartic acid (Asp/D), PpHSFA2a‐D, to mimic the phosphorylation state. (e) Effect of phosphorylation on the binding capacity of PpHSFA2a to HSE. The *** indicates significant differences at *P* < 0.001.

To verify whether PpHSFA2a can directly regulate the transcription of downstream target genes using a dual‐luciferase reporter (DLR) assay. The p35S‐PpHSFA2a‐PEAQ was as an effector, and the promoters of *PpHSP18.5*, *PpHSP70*, *PpGSTU7*, *PpGSTU19*, *PpGolS1* and *PpBAM1* were constructed into the PGREENII 0800‐LUC vector as a reporter (Figure 7a). The results showed that the LUC/REN ratios of PpHSFA2a combined with *PpHSP18.5*, *PpHSP70*, *PpGSTU7*, *PpGSTU19*, *PpGolS1* and *PpBAM1* were significantly higher than the control, indicating that PpHSFA2a can directly activate the transcription of these genes (Figure 7b). Subsequently, the electrophoretic mobility shift assay (EMSA) was used to verify whether PpHSFA2a could directly bind HSE in the promoters of these genes. In lane 1, the probe did not migrate without His‐PpHSFA2a. When the His‐PpHSFA2a protein was added to lane 2, the labelled probe could form a blocking band with the His‐PpHSFA2a protein. The band shifting was weakened upon the addition of increasing amounts of the cold probe in lanes 3 and 4. In lane 5, after adding the mutant labelled probe (AAAAAAAA), the mutant labelled probe could not bind to the His‐PpHSFA2a to form a blocking band (Figure 7c). These results indicated that PpHSFA2a can directly bind HSE in the promoters of *PpHSP18.5*, *PpHSP70*, *PpGSTU7*, *PpGSTU19*, *PpGolS1* and *PpBAM1* to regulate the transcription of these genes.

To investigate the effect of phosphorylation on the function of PpHSFA2a, the three ser phosphorylated sites of PpHSFA2a were mutated to alanine (PpHSFA2a‐A) to simulate the non‐phosphorylated state and mutated to aspartic acid (PpHSFA2a‐D) to mimic the phosphorylated state. The phosphorylation site is located in the NLS functional domain and may affect the subcellular localization of PpHSFA2a. Subcellular localization experiments showed that PpHSFA2a‐GFP was expressed in the cytoplasm and nucleus. Notably, most PpHSFA2a‐D‐GFP was expressed in the nucleus, while PpHSFA2a‐A‐GFP was mainly expressed in the cytoplasm (Figure 7d). These results demonstrate that the phosphorylation of Ser243, Ser245 and Ser247 in the NLS functional domain could transfer the localization of PpHSFA2a from the cytoplasm to the nucleus.

HSFs in the nucleus can form stable trimers that transcriptionally activate target genes in the nucleus (Kmiecik *et al*., 2020). PpHSFA2a was phosphorylated and then transferred to the nucleus. Next, we performed the EMSA analysis of the effect of phosphorylation‐mediated nuclear migration on the binding ability with HSE. The proteins of His‐PpHSFA2a, His‐PpHSFA2a‐D and His‐PpHSFA2a‐A were quantified using the BCA and incubated with labelled probes, respectively. Phosphorylated PpHSFA2a‐D has a deeper binding block with *PpHSP18.5*, *PpHSP70*, *PpGSTU7*, *PpGSTU19*, *PpGolS1* and *PpBAM1* (Figure 7e). In conclusion, the binding ability of PpHSFA2a‐D to DNA was significantly higher than that of PpHSFA2a and PpHSFA2a‐A, indicating that the phosphorylation of PpHSFA2a by PpCDPK29 also increased its binding ability to DNA.

## Discussion

Previous studies found that intracellular ROS were transiently and rapidly increased during the cold pre‐storage period after heat treatment in bananas, cucumbers, peaches and other fruits and vegetables, which would enhance the activity of the antioxidant enzyme system and improve the cold resistance of fruits and vegetables (Endo *et al*., 2019; Hu *et al*., 2021a; Huan *et al*., 2017; Wang *et al*., 2012). In addition to causing oxidative damage to plant tissues, ROS is also considered a common signal molecule in plant stress response signalling pathways, interacting with other signals to improve the cross‐tolerance of cells (Choudhury *et al*., 2013; Waszczak *et al*., 2018). The study found that the ROS level increased in the early cold storage after HW treatment, and the Ca^2+^ concentration also had a similar trend (Figure 1f,g). ROS is an early signal to activate Ca^2+^ and other signalling and can regulate the intensity of intracellular Ca^2+^ signalling. Meanwhile, Ca^2+^ can also activate plasma membrane NADPH oxidase to generate ROS, which leads to more cytoplasmic Ca^2+^ inward flow to form the Ca^2+^ signalling (Gapper and Dolan, 2006; Pei *et al*., 2000). However, HW + DPI treatment inhibited the production of ROS in the early stage, and the activities of SOD, CAT and APX were relatively low. With the storage time prolonged, the inhibitory effect of DPI pretreatment decreased. ROS burst may have occurred in the later stage, resulting in an imbalance of ROS homeostasis, making the oxidative stress more severe and aggravating the chilling injury in the last storage (Figure 1a–e). This indicates that HW treatment can activate ROS signalling, and ROS can regulate plasma membrane Ca^2+^ channels, increase cytoplasmic Ca^2+^ concentration, form the ROS‐Ca^2+^ signalling pathway and enhance antioxidant capacity.

CDPKs are at the crossover between Ca^2+^ and ROS signalling and play a central role in the Ca^2+^ –ROS signalling pathway by phosphorylating RBOHs (Kobayashi *et al*., 2012). HW treatment can activate ROS signalling through the RBOH pathway and activate Ca^2+^ signalling, suggesting that CDPK may play an important role in this process. Previous research demonstrated that CDPKs could phosphorylate the N‐terminal of the RBOH protein and participate in RBOH‐mediated ROS bursts (Dubiella *et al*., 2013; Zhao *et al*., 2021). For instance, StCDPK4 and StCDPK5 regulated ROS production by phosphorylating NADPH oxidase in potatoes (Kobayashi *et al*., 2012). Meanwhile, CDPKs can scavenge excessive ROS by regulating antioxidant enzyme‐related genes. AtCPK8 and OsCPK10 could interact with CAT (Bundo and Coca, 2017; Zou *et al*., 2015), and SlCPK28 could phosphorylate SlAPX2 (Hu *et al*., 2021b), indicating that CDPKs can directly participate in the production and clearance of ROS through protein interaction and phosphorylation. In this study, we found that PpCDPK29 interacts with PpRBOHD and PpRBOHC on the cell membrane and may respond to the change of Ca^2+^ signal on the plasma membrane and promote ROS production (Figure 3). At the same time, PpCDPK29 also interacts with antioxidant enzyme proteins PpSOD and PpCAT1 to scavenge excessive ROS accumulation (Figure 4). Therefore, HW could induce PpCDPK29 to participate in the production and removal of ROS, thereby maintaining the homeostasis of ROS during low temperature storage of peach fruit.

In this study, we found that heat and low temperatures treatment increased the expression of the *PpCDPK29* gene. Heterologous overexpression of the *PpCDPK29* could improve the cold resistance of transgenic tomatoes, whereas tomato plants and fruits knocked out of the *SlCDPK29* were more sensitive to cold stress (Figure 2c–g). These results suggested that PpCDPK29 acted as a positive regulator of chilling tolerance. Indeed, numerous studies have shown that CDPKs play a vital role in the cold response of various species. For instance, *OsCPK13* was induced by cold stress, and overexpression improved the freezing tolerance of rice (Abbasi *et al*., 2004). OsCPK24 enhanced cold resistance by phosphorylating OsGrx10 to maintain higher glutathione levels (Liu *et al*., 2018). Besides, SlCPK27 regulated cold stress by participating in ABA, ROS, NO and MPK signalling pathways (Lv *et al*., 2018).

It is worth noting that HSFs have a cross‐adaptation function, enabling plants to enhance their tolerance to other stresses under certain stresses (Jacob *et al*., 2017). AtHSFs and AtHSPs mediate a crossover between heat and cold stress regulatory networks in *Arabidopsis* (Swindell *et al*., 2007). Heat treatment induced the expression of EjHSF1, which responded to low temperature by transcriptional regulation of the HSP, reducing the chilling injury of loquat fruit (Zeng *et al*., 2016). Heat stress treatment improved cold tolerance by CsHSFA1‐activated JA biosynthesis and signalling pathways (Qi *et al*., 2022). Here, HW treatment significantly induced the expression of PpHSFA2a, indicating that PpHSFA2a might be involved in the cross‐reaction of HW treatment to reduce the chilling injury of peach fruit. Intriguingly, we found that PpCDPK29 could phosphorylate three serines in the NLS domain of PpHSFA2a and phosphorylation caused the transfer of PpHSFA2 to the nucleus (Figure 5e,f). The function of HSF is mainly to transform into a trimer state in the nucleus, and the HSF homotrimer binds to the HSE of the downstream gene promoter region and transcribes (Sakurai and Enoki, 2010). Studies have demonstrated that HSF can regulate downstream *HSP* and various stress‐related genes. In *Arabidopsis*, salt stress induced the expression of AtHSFA7b, and AtHSFA7b bound to HSEs to regulate its downstream target genes, such as *SOSs*, *NHXs*, *HPP2C5*, *RHC1*, *SODs*, *POD*s, *GSTs*, *P5CS*, *CESAs*, *CSLs* and some transcription factors to maintain the K^+^/Na^+^ balance of cells, improve the ability to scavenge ROS, enhance osmotic potential and reduce water loss, thereby improving the tolerance to salt stress (Zang *et al*., 2019). Herein, DLR and EMSA assays showed that PpHSFA2a could directly bind to the HSE in the promoters of *PpHSP18.5*, *PpHSP70*, *PpGSTU7*, *PpGSTU19*, *PpGolS1* and *PpBAM1* to activate gene transcription (Figure 7a–c). Furthermore, phosphorylation could enhance the binding ability of PpHSFA2a to the promoter HSE of downstream target genes (Figure 7e). Consequently, PpHSFA2a was phosphorylated by PpCDPK29, resulting in plasma‐nucleus translocation of PpHSFA2a, which enhanced the regulation of the target gene. These target genes could act as molecular chaperones to protective proteins, improve ROS clearance and enhance osmotic regulation to reduce postharvest chilling injury of peach fruit.

Based on the results from this study, we proposed a working model: heat treatment activated ROS signalling and regulated plasma membrane Ca^2+^ channels to activate Ca^2+^ signalling, and PpCDPK29 sensed Ca^2+^ signalling and then induced ROS production by interacting with RBOHC/D, forming a Ca^2+^ –ROS signalling pathway. Subsequently, PpCDPK29 could interact with PpSOD and PpCAT to activate the antioxidant system and remove excessive ROS, thereby maintaining the homeostasis of ROS. Meanwhile, PpCDPK29 could phosphorylate PpHSFA2a and enhance the regulation of PpHSFA2a on downstream defence‐related genes, thereby improving the chilling resistance of fruits (Figure S15). In conclusion, HW treatment alleviated postharvest chilling injury of peach fruit by activating PpCDPK29‐mediated Ca^2+^–ROS and HSF–HSP signalling pathways. These results preliminarily elucidate the signalling and molecular mechanisms of HW treatment to reduce chilling injury and provide a new tract for the breeding strategy to improve the chilling tolerance of tomato fruits for low‐temperature storage.

## Experimental procedures

### Material, treatments and chilling injury‐related indicators

The cold‐sensitive peach ‘Hujingmilu’ was harvested from the Jiangsu Academy of Agricultural Sciences in Nanjing. Fruits of similar size and ripeness were randomly divided into three groups, including the control (distilled water at room temperature for 10 min), HW treatment (soaking in 45 °C distilled water for 10 min) (Wang *et al*., 2022) and HW + DPI treatment (45 °C distilled water with 10 μmol L^−1^ DPI for 10 min). Fruits were dried naturally and stored in 0.01 mm thick polyethylene plastic bags at 5 ± 1 °C (90% relative humidity) for 28 days. There were three biological replicates for each treatment. Samples were collected at 0, 3, 6, 12, 24, 48, 72 h and 7, 14, 21, 28 days of cold storage.

Chilling injury‐related indicators were measured according to the method described in our previous study and described in Method S1 (Zhao *et al*., 2023).

### Detection of intracellular Ca^2+^ concentration, ROS levels and antioxidant enzyme activity

Intracellular free Ca^2+^ concentration was detected by fluorescence imaging using the Ca^2+^ fluorescent probe, Fluo‐3/AM, based on our previous study (Hou *et al*., 2023). The fruit slices were soaked in a 10 μM Fluo‐3 AM‐ester solution, incubated at 37 °C for 1 h in the dark and then washed three to five times with HEPES buffer. The Ca^2+^ fluorescence signal images were observed using the laser scanning confocal microscope LSM780 (Zeiss, Germany). Total ROS was detected using a fluorescent probe 2',7'‐dichlorodihydrofluorescein diacetate (H_2_DCF‐DA). The sarcocarp sections were stained in a 10 μM H_2_DCF‐DA solution for 30 min and then rinsed thrice with PBS buffer. Fluorescence images were observed using a fluorescence microscope at excitation/emission wavelengths of 485/525 nm. The relative fluorescence intensities were analysed and calculated using Image‐J software.

### RNA extraction and quantitative real‐time polymerase chain reaction (qRT‐PCR)

Total RNA was extracted using the Plant RNA Extraction Kit (Chengdu Fuji Biotechnology Co., Ltd.). About 1 μg of RNA was transcribed into cDNA using HiScript Q RT SuperMix for qPCR (+gDNA wiper) (YEASEN, China). qRT‐PCR was performed using Thermo QuantStudio 3 & QuantStudio 5 software with SYBR^®^ Green Premix kit (YEASEN, China) (primers listed in Table S1). Each sample was repeated three times, and the relative gene expression was calculated using the 2^−∆∆Ct^ method (Livak and Schmittgen, 2001). The methods for determining antioxidant enzyme activity are listed in Methods S2 (Zhao *et al*., 2023).

### Subcellular localization

The full length CDSs of PpCDPK29 and PpHSFA2a without stop codon were cloned from peach cDNA using 2 × Phanta Flash Master Mix (Vazyme, China) and constructed into the pCAMBIA1305 vector by ClonExpress II One Step Cloning Kit (Vazyme, China), forming the fusion expression vector (p35S::PpCDPK29‐GFP and p35S::PpHSFA2a ‐GFP) and transferred into GV3101 *Agrobacterium* competent cells. The infection solutions of 35S::PpCDPK29‐GFP, p35S::PpHSFA2a‐GFP and 35S::GFP were injected into the lower surface of 4‐week‐old *Nicotiana benthamiana* leaves. After 60 h of culture, green fluorescence was observed using the laser confocal microscope (Zeiss LSM 780, Germany).

### Cold stress treatment and tolerance assays of transgenic plants

The generation of transgenic tomatoes was described in Method S2. The WT and transgenic tomato plants with five leaves were exposed at 4 °C, and the control at 25 °C for 14 days. Transgenic and WT tomato fruit at the green ripening stage were harvested and stored in an incubator at 4 ± 1 °C and 80%–85% relative humidity. Tomato fruits were observed with CI symptoms for cold tolerance assays after 14 and 21 days of cold stress and 14 days of storage at room temperature. Samples were taken at 0 h, 12 h, 7 days, 14 days and 21 days. Colour parameter (a* value) was measured by a colorimeter (CR‐400). DAB and NBT staining methods were described in Methods S4. Transient expression in peach fruit was described in Methods S5 (Zhao *et al*., 2023).

### RNA‐seq assay

Samples of transgenic and WT tomato fruits stored at low temperatures for 12 h and 14 days were used for RNA sequencing (RNA‐Seq). Sequencing was performed on the Illumina NovaSeq platform, and raw reads were processed using the bioinformatics analysis platform BMKCloud (www.biocloud.net). Gene functions were annotated by sequence alignment using the Nr, Pfam, Swiss‐Prot, GO, COG/KOG and KEGG databases and gene expression levels were estimated by FPKM values.

### Metabolomics analysis

Liquid chromatography–tandem mass spectrometry (LCMS/MS) was used to determine the metabolites in transgenic and WT tomato fruits. Six biological replicates were performed for each sample. The detection platform was the Waters Acquity I‐Class PLUS ultra high performance liquid chromatography in tandem with the Waters Xevo G2‐XS QTOF high resolution mass spectrometer. The raw data collected by MassLynx V4.2 were identified based on the Progenesis QI software online METLIN database and public databases.

### Yeast two‐hybrid (Y2H) assay

Yeast two‐hybridization was performed using Clontech's Matchmaker GAL4 yeast two‐hybrid system, and the yeast strain was AH109. The plasmid vectors used were pGADT7, pGBKT7, pGBKT7‐P53, pGADT7‐T and pGBKT7‐Lam. The positive control was co‐transfection of pGADT7‐T and pGBKT7‐P53. The negative control was co‐transfected with pGADT7‐T and pGBKT7‐Lam. The full‐length CDSs of PpRBOHD, PpRBOHC, PpSOD, PpCAT1 and PpHSFA2a were constructed into the pGADT7 vector (primers listed in Table S1) and co‐transfected with PpCDPK29‐BK into AH109 on SD/‐Trp‐/‐Leu medium and then validated on SD/‐Leu/‐Trp/‐His/‐Ade and SD/‐Leu/‐Trp/‐His/‐Ade/X‐*α*‐gal selective medium.

### Luciferase complementation imaging (LCI) assay

The PpCDPK29 was constructed into the pCAMBIA1300‐nLUC vector, and the PpCDPK29 interacting proteins were connected to the pCAMBIA1300‐cLUC vector (primers in Table S1). Fusion expression vectors of PpCDPK29‐nLUC, PpRBOHD‐cLUC, PpRBOHC‐cLUC, PpSOD‐cLUC and PpCAT1‐cLUC were obtained and transferred into *Agrobacterium* GV3101 competent cells. *Agrobacterium* transient infestation was used, and 48 h after infestation, 1 mM D‐fluorescein potassium salt was sprayed on the back of *Nicotiana benthamiana* leaves and placed in a dark environment for 5 min. The luminescence was detected and photographed using the plant NightSHADE LB 985 imaging system (Berthold) (Zhao *et al*., 2022).

### Bimolecular fluorescence complementation (BiFC) assays

The CDS of PpCDPK29 was constructed into the pSPYNE vector, and the CDS of PpRBOHD, PpRBOHC, PpSOD, PpCAT1 and PpHSFA2a was connected to the pSPYCE vector. The constructed fusion vectors were transformed into *Agrobacterium tumefaciens* GV3101 to infect *Nicotiana benthamiana* leaves. After 48–72 h of culture, the YFP fluorescence of tobacco leaves was observed by LSM780 laser confocal microscopy (Zeiss, Germany).

### In vitro GST pull‐down assay

The full‐length CDSs of PpCDPK29, PpRBOHD, PpRBOHC, PpSOD, PpCAT1 and PpHSFA2a were cloned and formed fusion vectors with GST or His tag, and recombinant proteins were expressed and purified with HisSep Ni‐NTA Agarose Resin 6FF or GSTSep Glutathione Agarose Resin 4FF (YEASEN, China). The GST‐tagged protein was bound to glutathione agarose resin, and then the interacting protein containing the His tag was added. The proteins were shaken on a horizontal shaker at 4 °C for 4 h and then washed three times with PBS buffer containing 1% Triton‐100. Finally, the bound proteins were eluted and detected by 10% SDS‐PAGE. Western blot (WB) assay was performed using the one‐step rapid WB kit (CW2030M, CWBIO, China).

### Dual‐luciferase reporter (DLR) assay

The promoter sequences of *PpHSP18.5*, *PpHSP70*, *PpGSTU7*, *PpGSTU19*, *PpGolS1* and *PpBAM1* were cloned from peach DNA and connected to the PGREENII 0800‐LUC reporter vector as a reporter. The p35S‐PpHSFA2a‐PEAQ recombinant plasmid was used as an effector, and the PEAQ empty vector was used as the control. The fusion plasmids were transferred into *Agrobacterium* GV3101 competent cells. The effector and reporter were mixed at an 8:1 ratio and injected into tobacco leaves (Zhao *et al*., 2023). After a 60 h injection, the LUC/REN ratio was measured using the dual luciferase kit (DL101‐01, Vazyme, China).

### Electrophoretic mobility shift assay (EMSA)

EMSA assay was carried out according to the previous method (Hou *et al*., 2023) and performed with His‐PpHSFA2a (without stop codon) recombinant protein. The 50 bp fragment containing HSE (HSEs: 5'‐GAANNTTC‐3') elements on the promoters of *PpHSP18.5*, *PpHSP70*, *PpGSTU7*, *PpGSTU19*, *PpGolS1* and *PpBAM1* was synthesized as the probe. The probe was labelled with the probe biotin labeling kit (Beyotime, China). The unlabeled sequence was used as a competitive cold probe, and the HSE was mutated to the AAAAAAA sequence as a mutant probe. EMSA was performed using the chemiluminescent EMSA kit (GS009, Beyotime, China). The protein‐DNA complex was separated by 6% polyacrylamide gel and visualized on a ChemiDoc™ MP Imaging System (Bio‐Rad, Hercules, CA, USA) instrument.

### Phos‐tag SDS‐PAGE phosphorylation assay in vitro and mass spectrometry

The phosphorylation assay was performed according to the Phos‐tag™ SDS‐PAGE (APExBIO, US) protocol instructions. The 50 μL of kinase reaction system contained 10 μL of His‐PpHSFA2a, 5 μL of GST‐PpCDPK29, 5 μL of 10 × kinase reaction buffer (250 mM Tris–HCl pH 7.5100 mM MgCl_2_,10 mM CaCl_2_,10 mM ATP, 10 mM DTT), 5 μL of protease inhibitor, and 25 μL of H_2_O. The negative control was the addition of calf intestinal alkaline phosphatase (CIAP) for a dephosphorylated state. The kinase reaction was carried out at 30 °C for 30 min, and 20 μL of the reaction product was detected for phosphorylation by Phos‐tag SDS‐PAGE. The phosphorylated proteins were analysed using liquid chromatography tandem mass spectrometry (LC–MS/MS).

### Targeted gene mutation assay

PpHSFA2a gene‐targeted mutation assay was performed using the Mut Express II Fast Mutagenesis Kit V2 (C214, Vazyme, China) kit. Primers containing site‐directed mutagenesis 15 bp reverse complementary region and 15 bp non‐complementary region were designed. After inverse PCR amplification of the plasmid, 1 μL of *Dpn*I was added to the reaction at 37 °C for 2 h to digest the amplified product, remove the methylated template plasmid, and then use the recombinant reaction to achieve the in vitro cyclization of linear DNA, transformed into the *E. coli* competent cells for sequencing to identify mutations.

### Statistical analysis

The data were expressed as mean ± standard error (SE) and statistically analysed by GraphPad Prism and SPSS 22.0. The *P*‐value <0.01 or 0.05 was considered statistically significant.

## Conflict of interest

The authors declare no conflict of interest.

## Author contributions

P.J. and Y.H.Z. designed and managed the research. L.Y.Z. contributed to the experimental design and management, data analysis and manuscript writing. L.Y.Z. and Y.Q.Z. performed the experiments. Y.Q.B., Y.Y.H. and Y.L. prepared the plant materials and laboratory equipment during the experiment. H.W., M.B. and Z.G.W. provided suggestions and reviewed and modified this manuscript.

## Supporting information


**Figure S1** Effects of HW and HW + DPI treatments on ROS and Ca^2+^ fluorescence and relative fluorescence intensity of peach fruit. (a‐b) ROS and Ca^2+^ relative fluorescence intensity of peach fruit in the early cold storage stage. (c‐d) ROS and Ca^2+^ concentration fluorescence during cold‐store peach fruit. (e) Relative fluorescence intensity of Ca^2+^ concentration during cold storage of peach fruit.
**Figure S2** Characterization of PpCDPK29. (a) Protein structural characteristics of PpCDPK29. (b) Multiple alignments of amino acid sequence of PpCDPK29.
**Figure S3** Identification of transgenic tomato positive seedlings. (a) The expression of *PpCDPK29* in positive plants of T1 generation was detected by semi‐quantitative PCR. M: DNA marker. (b) Relative gene expression of *PpCDPK29* in tomato T1‐generation positive seedlings. (c) Sequencing results of CRISPR/Cas9 tomato mutation lines.
**Figure S4** Analysis of DEGs between RNA‐Seq groups. (a) PCA of RNA‐Seq samples. (b) DEGs distribution histogram. (c) 12 h and (d) 14 days Venn diagrams of DEGs.
**Figure S5** GO and KEGG analysis of DEGs in WT and transgenic tomato fruits. (a) 12 h and (b) 14 days enrichment analysis of DEGs in the ‘biological process’ GO subcategories. (c) and (d) KEGG analysis of DEGs in WT and transgenic tomato fruits.
**Figure S6** Metabolome differential metabolite analysis of WT and transgenic tomato fruits. (a) Correlation coefficient of each sample. (b) DEMs distribution histogram. (c) KEGG analysis of DEMs.
**Figure S7** Cross‐comparisons between transcriptomic and metabolome. (a) Venn diagram of DEGs and DEMs pathways by cross‐comparisons between transcriptomic and metabolome. (b) Heat map of DEGs in Glutathione metabolism, Starch and sucrose metabolism and Galactose metabolism pathway.
**Figure S8** PpCDPK29 yeast two‐hybrid vector construction. (a) Yeast two‐hybrid protein fragment of PpCDPK29. (b) Validation of yeast self‐activation of PpCDPK29.
**Figure S9** Gene expression analysis of *PpCAT1* (a) and *PpSOD* (b).
**Figure S10** Identification and protein characterization of PpHSFA2a. (a) Phylogeny and amino acid multiple sequence analysis of the HSFA2 in peach and other species. (b) The relative expression of *PpHSFA2a* under HW treatment during cold storage.
**Figure S11** Effects of CaCl_2_ and EGTA treatment on the interaction between PpCDPK29 and PpHSFA2a.
**Figure S12** Effect of temperature on subcellular localization of PpHSFA2a (Bars: 100 μm). Under normal temperature conditions, PpHSFA2a was located in the cytoplasm and nucleus, but under heat stress 37 °C for 4 h, PpHSFA2a was more transferred to the nucleus, and most of it was localized in the nucleus. However, under 4 h of low‐temperature treatment at 4 °C, the localization change was not obvious, compared with normal temperature, only a small part was transferred to the nucleus.
**Figure S13** Expression level analysis of PpHSFA2a target gene. (a‐f) The relative expression levels of *PpHSP18.5* (a), *PpHSP70* (b), *PpGSTU7* (c), *PpGSTU19* (d), *PpGolS1* (e) and *PpBAM1* (f).
**Figure S14** Transient overexpression and silencing analysis of *PpHSFA2a*. (a) The schematic diagram of *Agrobacterium* infiltration of peach fruits. (b) Fluorescence expression of GFP in peach fruit under transient infection. (c) The expression levels of *PpHSFA2a* after overexpression *PpHSFA2a*.
**Figure S15** Proposed model for the PpCDPK29 signalling cascade involved in peach fruit chilling injury. HW treatment activates PpCDPK29. Then, PpCDPK29 interacts with RBOHC/D to activate ROS, and interacts with SOD and CAT to scavenge excess ROS, thus maintaining ROS homeostatic balance. Meanwhile, PpCDPK29 can phosphorylate PpHSFA2a, and PpHSFA2a can directly regulate downstream defence‐related genes alleviating chilling injury.
**Method S1** Measurement of chilling injury‐related indicators.
**Method S2** SOD, CAT and APX activities measurement.
**Method S3** Generation of transgenic plants.
**Method S4** DAB and NBT staining.
**Method S5** Transient expression in peach fruit.

**Table S1** The sequence of primers used in this study.

## Data Availability

Data supporting the results of this study can be found in the supplementary materials of this article.

## References

[pbi70024-bib-0001] Abbasi, F. , Onodera, H. , Toki, S. , Tanaka, H. and Komatsu, S. (2004) OsCDPK13, a calcium‐dependent protein kinase gene from rice, is induced by cold and gibberellin in rice leaf sheath. Plant Mol. Biol. 55, 541–552. 15604699 10.1007/s11103-004-1178-y

[pbi70024-bib-0002] Åkerfelt, M. , Morimoto, R.I. and Sistonen, L. (2010) Heat shock factors: integrators of cell stress, development and lifespan. Nat. Rev. Mol. Cell Biol. 11, 545–555. 20628411 10.1038/nrm2938 PMC3402356

[pbi70024-bib-0003] Asano, T. , Hayashi, N. , Kikuchi, S. and Ohsugi, R. (2012a) CDPK‐mediated abiotic stress signaling. Plant Signal. Behav. 7, 817–821. 22751324 10.4161/psb.20351 PMC3583972

[pbi70024-bib-0004] Asano, T. , Hayashi, N. , Kobayashi, M. , Aoki, N. , Miyao, A. , Mitsuhara, I. , Ichikawa, H. *et al*. (2012b) A rice calcium‐dependent protein kinase OsCPK12 oppositely modulates salt‐stress tolerance and blast disease resistance. Plant J. 69, 26–36. 21883553 10.1111/j.1365-313X.2011.04766.x

[pbi70024-bib-0005] Bokhary, S.U.F. , Wang, L. , Zheng, Y. and Jin, P. (2020) Pre‐storage hot water treatment enhances chilling tolerance of zucchini (*Cucurbita pepo* L.) squash by regulating arginine metabolism. Postharvest Biol. Technol. 166, 111229.

[pbi70024-bib-0006] Bundo, M. and Coca, M. (2017) Calcium‐dependent protein kinase OsCPK10 mediates both drought tolerance and blast disease resistance in rice plants. J. Exp. Bot. 68, 2963–2975. 28472292 10.1093/jxb/erx145 PMC5853374

[pbi70024-bib-0007] Campo, S. , Baldrich, P. , Messeguer, J. , Lalanne, E. , Coca, M. and San Segundo, B. (2014) Overexpression of a calcium‐dependent protein kinase confers salt and drought tolerance in rice by preventing membrane lipid peroxidation. Plant Physiol. 165, 688–704. 24784760 10.1104/pp.113.230268 PMC4044838

[pbi70024-bib-0008] Cheng, S.H. , Willmann, M.R. , Chen, H.C. and Sheen, J. (2002) Calcium signaling through protein kinases. The Arabidopsis calcium‐dependent protein kinase gene family. Plant Physiol. 129, 469–485. 12068094 10.1104/pp.005645 PMC1540234

[pbi70024-bib-0009] Choudhury, S. , Panda, P. , Sahoo, L. and Panda, S.K. (2013) Reactive oxygen species signaling in plants under abiotic stress. Plant Signal. Behav. 8, e23681. 23425848 10.4161/psb.23681 PMC7030282

[pbi70024-bib-0010] Crisosto, C.H. , Johnson, R.S. , DeJong, T. and Day, K.R. (1997) Orchard factors affecting postharvest stone fruit quality. HortScience. 32, 820–823.

[pbi70024-bib-0011] Ding, Y. , Yang, H. , Wu, S. , Fu, D. , Li, M. , Gong, Z. and Yang, S. (2022) CPK28‐NLP7 module integrates cold‐induced Ca^2+^ signal and transcriptional reprogramming in Arabidopsis. Sci. Adv. 8, eab7901. 10.1126/sciadv.abn7901 PMC9242591 35767615

[pbi70024-bib-0012] Dubiella, U. , Seybold, H. , Durian, G. , Komander, E. , Lassig, R. , Witte, C.P. , Schulze, W.X. *et al*. (2013) Calcium‐dependent protein kinase/NADPH oxidase activation circuit is required for rapid defense signal propagation. Proc. Natl. Acad. Sci. U. S. A. 110, 8744–8749. 23650383 10.1073/pnas.1221294110 PMC3666735

[pbi70024-bib-0013] Endo, H. , Miyazaki, K. , Ose, K. and Imahori, Y. (2019) Hot water treatment to alleviate chilling injury and enhance ascorbate‐glutathione cycle in sweet pepper fruit during postharvest cold storage. Sci. Hortic. 257, 108715.

[pbi70024-bib-0014] Gapper, C. and Dolan, L. (2006) Control of plant development by reactive oxygen species. Plant Physiol. 141, 341–345. 16760485 10.1104/pp.106.079079 PMC1475470

[pbi70024-bib-0015] Guo, M. , Liu, J.H. , Ma, X. , Luo, D.X. , Gong, Z.H. and Lu, M.H. (2016) The plant heat stress transcription factors (HSFs): Structure, regulation, and function in response to abiotic stresses. Front. Plant Sci. 7, 114. 26904076 10.3389/fpls.2016.00114 PMC4746267

[pbi70024-bib-0016] Haider, S. , Raza, A. , Iqbal, J. , Shaukat, M. and Mahmood, T. (2022) Analyzing the regulatory role of heat shock transcription factors in plant heat stress tolerance: a brief appraisal. Mol. Biol. Rep. 49, 5771–5785. 35182323 10.1007/s11033-022-07190-x

[pbi70024-bib-0017] Hoang, T.V. , Vo, K.T.X. , Rahman, M.M. , Choi, S.H. and Jeon, J.S. (2019) Heat stress transcription factor OsSPL7 plays a critical role in reactive oxygen species balance and stress responses in rice. Plant Sci. 289, 110273. 31623772 10.1016/j.plantsci.2019.110273

[pbi70024-bib-0018] Hou, Y. , Liu, Y. , Zhao, L. , Zhao, Y. , Wu, Z. , Zheng, Y. and Jin, P. (2023) EjCML19 and EjWRKY7 synergistically function in calcium chloride‐alleviated chilling injury of loquat fruit. Postharvest Biol. Technol. 203, 112417.

[pbi70024-bib-0019] Hu, J. , Zhang, M. , Li, J. , Gai, X. , Ling, Y. , Zheng, K. , Zhao, H. *et al*. (2021a) Effect of delay between hot water treatment and cold storage on quality and antioxidant enzyme system in cool‐stored “shenqing” cucumber. J. Food Process. Pres 45, 45.

[pbi70024-bib-0020] Hu, Z. , Li, J. , Ding, S. , Cheng, F. , Li, X. , Jiang, Y. , Yu, J. *et al*. (2021b) The protein kinase CPK28 phosphorylates ascorbate peroxidase and enhances thermotolerance in tomato. Plant Physiol. 186, 1302–1317. 33711164 10.1093/plphys/kiab120 PMC8195530

[pbi70024-bib-0021] Huan, C. , Han, S. , Jiang, L. , An, X. , Yu, M. , Xu, Y. , Ma, R. *et al*. (2017) Postharvest hot air and hot water treatments affect the antioxidant system in peach fruit during refrigerated storage. Postharvest Biol. Technol. 126, 1–14.

[pbi70024-bib-0022] Jacob, P. , Hirt, H. and Bendahmane, A. (2017) The heat‐shock protein/chaperone network and multiple stress resistance. Plant Biotechnol. J. 15, 405–414. 27860233 10.1111/pbi.12659 PMC5362687

[pbi70024-bib-0023] Jin, P. , Zheng, Y. , Tang, S. , Rui, H. and Wang, C.Y. (2009) A combination of hot air and methyl jasmonate vapor treatment alleviates chilling injury of peach fruit. Postharvest Biol. Tec. 52, 24–29.

[pbi70024-bib-0056] Kmiecik, S.W. , Le Breton, L. and Mayer, M.P. (2020) Feedback regulation of heat shock factor 1 (Hsf1) activity by Hsp70‐mediated trimer unzipping and dissociation from DNA. The EMBO journal 39, e104096. 32490574 10.15252/embj.2019104096 PMC7360973

[pbi70024-bib-0024] Kobayashi, M. , Yoshioka, M. , Asai, S. , Nomura, H. , Kuchimura, K. , Mori, H. , Doke, N. *et al*. (2012) StCDPK5 confers resistance to late blight pathogen but increases susceptibility to early blight pathogen in potato via reactive oxygen species burst. New Phytol. 196, 223–237. 22783903 10.1111/j.1469-8137.2012.04226.x

[pbi70024-bib-0025] Lauxmann, M.A. , Borsani, J. , Osorio, S. , Lombardo, V.A. , Budde, C.O. , Bustamante, C.A. , Monti, L.L. *et al*. (2014) Deciphering the metabolic pathways influencing heat and cold responses during post‐harvest physiology of peach fruit. Plant Cell Environ. 37, 601–616. 23937123 10.1111/pce.12181

[pbi70024-bib-0026] Li, L. , Wang, X. , Lv, J. , Duan, W. , Huang, T. , Zhao, K. , Meng, L. *et al*. (2023) Overexpression of sly‐miR167a delayed postharvest chilling injury of tomato fruit under low temperature storage. Postharvest Biol. Technol. 204, 112420.

[pbi70024-bib-0027] Liu, Y. , Xu, C. , Zhu, Y. , Zhang, L. , Chen, T. , Zhou, F. , Chen, H. *et al*. (2018) The calcium‐dependent kinase OsCPK24 functions in cold stress responses in rice. J. Integr. Plant Biol. 60, 173–188. 29193704 10.1111/jipb.12614

[pbi70024-bib-0028] Livak, K.J. and Schmittgen, T.D. (2001) Analysis of relative gene expression data using real‐time quantitative PCR and the 2^− ΔΔCT^ method. Methods 25, 402–408. 11846609 10.1006/meth.2001.1262

[pbi70024-bib-0029] Lurie, S. and Crisosto, C.H. (2005) Chilling injury in peach and nectarine. Postharvest Biol. Technol. 37, 195–208.

[pbi70024-bib-0030] Lv, X. , Li, H. , Chen, X. , Xiang, X. , Guo, Z. , Yu, J. and Zhou, Y. (2018) The role of calcium‐dependent protein kinase in hydrogen peroxide, nitric oxide and ABA‐dependent cold acclimation. J. Exp. Bot. 69, 4127–4139. 29868714 10.1093/jxb/ery212 PMC6054180

[pbi70024-bib-0031] Mittler, R. , Zandalinas, S.I. , Fichman, Y. and Van Breusegem, F. (2022) Reactive oxygen species signalling in plant stress responses. Nat. Rev. Mol. Cell Biol. 23, 663–679. 35760900 10.1038/s41580-022-00499-2

[pbi70024-bib-0032] Olate, E. , Jiménez‐Gómez, J.M. , Holuigue, L. and Salinas, J. (2018) NPR1 mediates a novel regulatory pathway in cold acclimation by interacting with HSFA1 factors. Nat. Plants. 4, 811–823. 30250280 10.1038/s41477-018-0254-2

[pbi70024-bib-0033] Pei, Z.‐M. , Murata, Y. , Benning, G. , Thomine, S. , Klüsener, B. , Allen, G.J. , Grill, E. *et al*. (2000) Calcium channels activated by hydrogen peroxide mediate abscisic acid signalling in guard cells. Nature 406, 731–734. 10963598 10.1038/35021067

[pbi70024-bib-0034] Pons, C. , Martí, C. , Forment, J. , Crisosto, C.H. , Dandekar, A.M. and Granell, A. (2014) A bulk segregant gene expression analysis of a peach population reveals components of the underlying mechanism of the fruit cold response. PLoS One 9, e90706. 24598973 10.1371/journal.pone.0090706 PMC3944608

[pbi70024-bib-0035] Qi, C. , Dong, D. , Li, Y. , Wang, X. , Guo, L. , Liu, L. , Dong, X. *et al*. (2022) Heat shock‐induced cold acclimation in cucumber through CsHSFA1d‐activated JA biosynthesis and signaling. Plant J. 111, 85–102. 35436390 10.1111/tpj.15780

[pbi70024-bib-0036] Ranty, B. , Aldon, D. , Cotelle, V. , Galaud, J.P. , Thuleau, P. and Mazars, C. (2016) Calcium sensors as key hubs in plant responses to biotic and abiotic stresses. Front. Plant Sci. 7, 327. 27014336 10.3389/fpls.2016.00327 PMC4792864

[pbi70024-bib-0037] Saijo, Y. , Hata, S. , Kyozuka, J. , Shimamoto, K. and Izui, K. (2000) Over‐expression of a single Ca^2+^‐dependent protein kinase confers both cold and salt/drought tolerance on rice plants. Plant J. 23, 319–327. 10929125 10.1046/j.1365-313x.2000.00787.x

[pbi70024-bib-0038] Sakurai, H. and Enoki, Y. (2010) Novel aspects of heat shock factors: DNA recognition, chromatin modulation and gene expression. FEBS J. 277, 4140–4149. 20945530 10.1111/j.1742-4658.2010.07829.x

[pbi70024-bib-0039] Shao, X. , Zhu, Y. , Cao, S. , Wang, H. and Song, Y. (2013) Soluble sugar content and metabolism as related to the heat‐induced chilling tolerance of loquat fruit during cold storage. Food Bioproc. Tech. 6, 3490–3498.

[pbi70024-bib-0040] Si, J. , Fan, Y.Y. , Liu, Z.L. , Wei, W. , Xiao, X.M. , Yang, Y.Y. , Shan, W. *et al*. (2022) Comparative transcriptomic analysis reveals the potential mechanism of hot water treatment alleviated‐chilling injury in banana fruit. Food Res. Int. 157, 111296. 35761601 10.1016/j.foodres.2022.111296

[pbi70024-bib-0041] Steinhorst, L. and Kudla, J. (2013) Calcium and reactive oxygen species rule the waves of signaling. Plant Physiol. 163, 471–485. 23898042 10.1104/pp.113.222950 PMC3793029

[pbi70024-bib-0042] Swindell, W.R. , Huebner, M. and Weber, A.P. (2007) Transcriptional profiling of Arabidopsis heat shock proteins and transcription factors reveals extensive overlap between heat and non‐heat stress response pathways. BMC Genomics 8, 1–15. 17519032 10.1186/1471-2164-8-125 PMC1887538

[pbi70024-bib-0043] Wang, H. , Zhang, Z. , Xu, L. , Huang, X. and Pang, X. (2012) The effect of delay between heat treatment and cold storage on alleviation of chilling injury in banana fruit. J. Sci. Food Agric. 92, 2624–2629. 22495636 10.1002/jsfa.5676

[pbi70024-bib-0044] Wang, X. , Zhuang, L. , Shi, Y. and Huang, B. (2017) Up‐regulation of HSFA2c and HSPs by ABA contributing to improved heat tolerance in tall fescue and arabidopsis. Int. J. Mol. Sci. 18(9), 1981. 28914758 10.3390/ijms18091981 PMC5618630

[pbi70024-bib-0045] Wang, L. , Hou, Y. , Wang, Y. , Hu, S. , Zheng, Y. and Jin, P. (2022) Genome‐wide identification of heat shock transcription factors and potential role in regulation of antioxidant response under hot water and glycine betaine treatments in cold‐stored peaches. J. Sci. Food Agric. 102, 628–643. 34146341 10.1002/jsfa.11392

[pbi70024-bib-0046] Waszczak, C. , Carmody, M. and Kangasjärvi, J. (2018) Reactive oxygen species in plant signaling. Annu. Rev. Plant Biol. 69, 209–236. 29489394 10.1146/annurev-arplant-042817-040322

[pbi70024-bib-0047] Wei, S. , Hu, W. , Deng, X. , Zhang, Y. , Liu, X. , Zhao, X. , Luo, Q. *et al*. (2014) A rice calcium‐dependent protein kinase OsCPK9 positively regulates drought stress tolerance and spikelet fertility. BMC Plant Biol. 14, 1–13. 10.1186/1471-2229-14-133 PMC4036088 24884869

[pbi70024-bib-0048] Yang, S. , Cai, W. , Shen, L. , Cao, J. , Liu, C. , Hu, J. , Guan, D. *et al*. (2022) A CaCDPK29‐CaWRKY27b module promotes CaWRKY40‐mediated thermotolerance and immunity to *Ralstonia solanacearum* in pepper. New Phytol. 233, 1843–1863. 34854082 10.1111/nph.17891

[pbi70024-bib-0049] Zang, D. , Wang, J. , Zhang, X. , Liu, Z. and Wang, Y. (2019) Arabidopsis heat shock transcription factor HSFA7b positively mediates salt stress tolerance by binding to an E‐box‐like motif to regulate gene expression. J. Exp. Bot. 70, 5355–5374. 31145794 10.1093/jxb/erz261 PMC6793466

[pbi70024-bib-0050] Zeng, J.K. , Li, X. , Zhang, J. , Ge, H. , Yin, X.R. and Chen, K.S. (2016) Regulation of loquat fruit low temperature response and lignification involves interaction of heat shock factors and genes associated with lignin biosynthesis. Plant Cell Environ. 39, 1780–1789. 27006258 10.1111/pce.12741

[pbi70024-bib-0051] Zhang, N. , Zhao, H. , Shi, J. , Wu, Y. and Jiang, J. (2020) Functional characterization of class I SlHSP17.7 gene responsible for tomato cold‐stress tolerance. Plant Sci. 298, 110568. 32771169 10.1016/j.plantsci.2020.110568

[pbi70024-bib-0052] Zhao, Y. , Du, H. , Wang, Y. , Wang, H. , Yang, S. , Li, C. , Chen, N. *et al*. (2021) The calcium‐dependent protein kinase ZmCDPK7 functions in heat‐stress tolerance in maize. J. Integr. Plant Biol. 63, 510–527. 33331695 10.1111/jipb.13056

[pbi70024-bib-0053] Zhao, L. , Xie, B. , Hou, Y. , Zhao, Y. , Zheng, Y. and Jin, P. (2022) Genome‐wide identification of the CDPK gene family reveals the CDPK‐RBOH pathway potential involved in improving chilling tolerance in peach fruit. Plant Physiol. Biochem. 191, 10–19. 36174282 10.1016/j.plaphy.2022.09.015

[pbi70024-bib-0054] Zhao, L. , Zhao, Y. , Wang, L. , Hou, Y. , Bao, Y. , Jia, Z. , Zheng, Y. *et al*. (2023) Hot water treatment improves peach fruit cold resistance through PpHSFA4c‐mediated HSF‐HSP and ROS pathways. Postharvest Biol. Technol. 199, 112272.

[pbi70024-bib-0055] Zou, J.J. , Li, X.D. , Ratnasekera, D. , Wang, C. , Liu, W.X. , Song, L.F. , Zhang, W.Z. *et al*. (2015) Arabidopsis CALCIUM‐DEPENDENT PROTEIN KINASE8 and CATALASE3 function in abscisic acid‐mediated signaling and H_2_O_2_ homeostasis in stomatal guard cells under drought stress. Plant Cell 27, 1445–1460. 25966761 10.1105/tpc.15.00144 PMC4456645

