# Supplementary material for: Calcium‐dependent protein kinase PpCDPK29‐mediated Ca2+‐ROS signal and PpHSFA2a phosphorylation regulate postharvest chilling tolerance of peach fruit
Source: Plant Biotechnol J. 2025 Feb 27;23(6):1938–53. doi: 10.1111/pbi.70024 (PMC12120869; doi:10.1111/pbi.70024)
Supplement: Supplementary file 1 — Figure S1 Effects of HW and HW + DPI treatments on ROS and Ca2+ fluorescence and relative fluorescence intensity of peach fruit. (a‐b) ROS and Ca2+ relative fluorescence intensity of peach fruit in the early cold storage stage. (c‐d) ROS and Ca2+ concentration fluorescence during cold‐store peach fruit. (e) Relative fluorescence intensity of Ca2+ concentration during cold storage of peach fruit. Figure S2 Characterization of PpCDPK29. (a) Protein structural characteristics of PpCDPK29. (b) Multiple alignments of amino acid sequence of PpCDPK29. Figure S3 Identification of transgenic tomato positive seedlings. (a) The expression of PpCDPK29 in positive plants of T1 generation was detected by semi‐quantitative PCR. M: DNA marker. (b) Relative gene expression of PpCDPK29 in tomato T1‐generation positive seedlings. (c) Sequencing results of CRISPR/Cas9 tomato mutation lines. Figure S4 Analysis of DEGs between RNA‐Seq groups. (a) PCA of RNA‐Seq samples. (b) DEGs distribution histogram. (c) 12 h and (d) 14 days Venn diagrams of DEGs. Figure S5 GO and KEGG analysis of DEGs in WT and transgenic tomato fruits. (a) 12 h and (b) 14 days enrichment analysis of DEGs in the ‘biological process’ GO subcategories. (c) and (d) KEGG analysis of DEGs in WT and transgenic tomato fruits. Figure S6 Metabolome differential metabolite analysis of WT and transgenic tomato fruits. (a) Correlation coefficient of each sample. (b) DEMs distribution histogram. (c) KEGG analysis of DEMs. Figure S7 Cross‐comparisons between transcriptomic and metabolome. (a) Venn diagram of DEGs and DEMs pathways by cross‐comparisons between transcriptomic and metabolome. (b) Heat map of DEGs in Glutathione metabolism, Starch and sucrose metabolism and Galactose metabolism pathway. Figure S8 PpCDPK29 yeast two‐hybrid vector construction. (a) Yeast two‐hybrid protein fragment of PpCDPK29. (b) Validation of yeast self‐activation of PpCDPK29. Figure S9 Gene expression analysis of PpCAT1 (a) and PpSOD (b). Figure S10 [file PBI-23-1938-s002.docx]

**Supplementary Data**

**Supplementary figure**


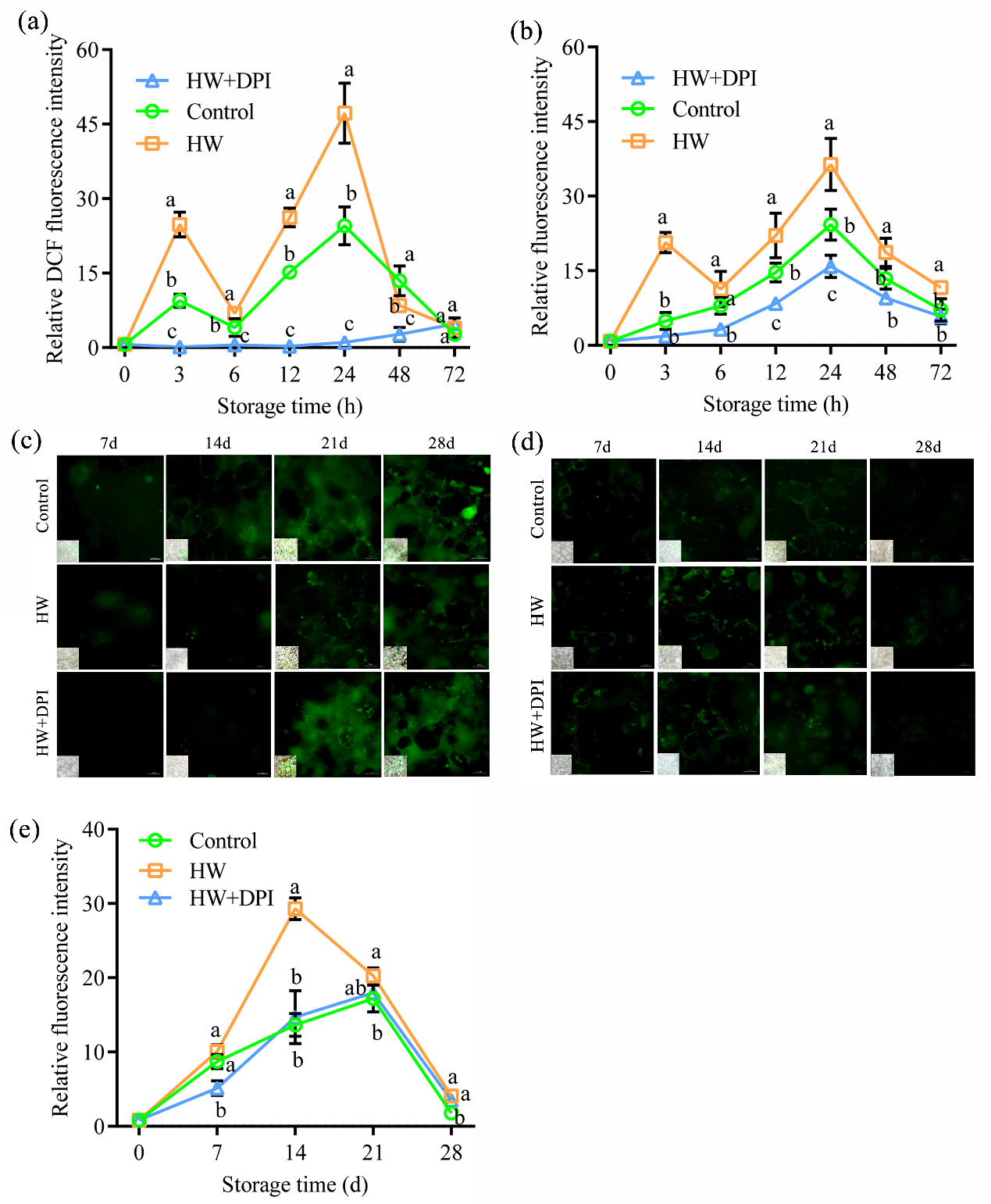


**Supplemental Figure S1** Effects of HW and HW+DPI treatments on ROS and Ca^2+^ fluorescence and relative fluorescence intensity of peach fruit. (a-b) ROS and Ca^2+^ relative fluorescence intensity of peach fruit in the early cold storage stage. (c-d) ROS and Ca^2+^ concentration fluorescence during cold-store peach fruit. (e) Relative fluorescence intensity of Ca^2+^ concentration during cold-store peach fruit.


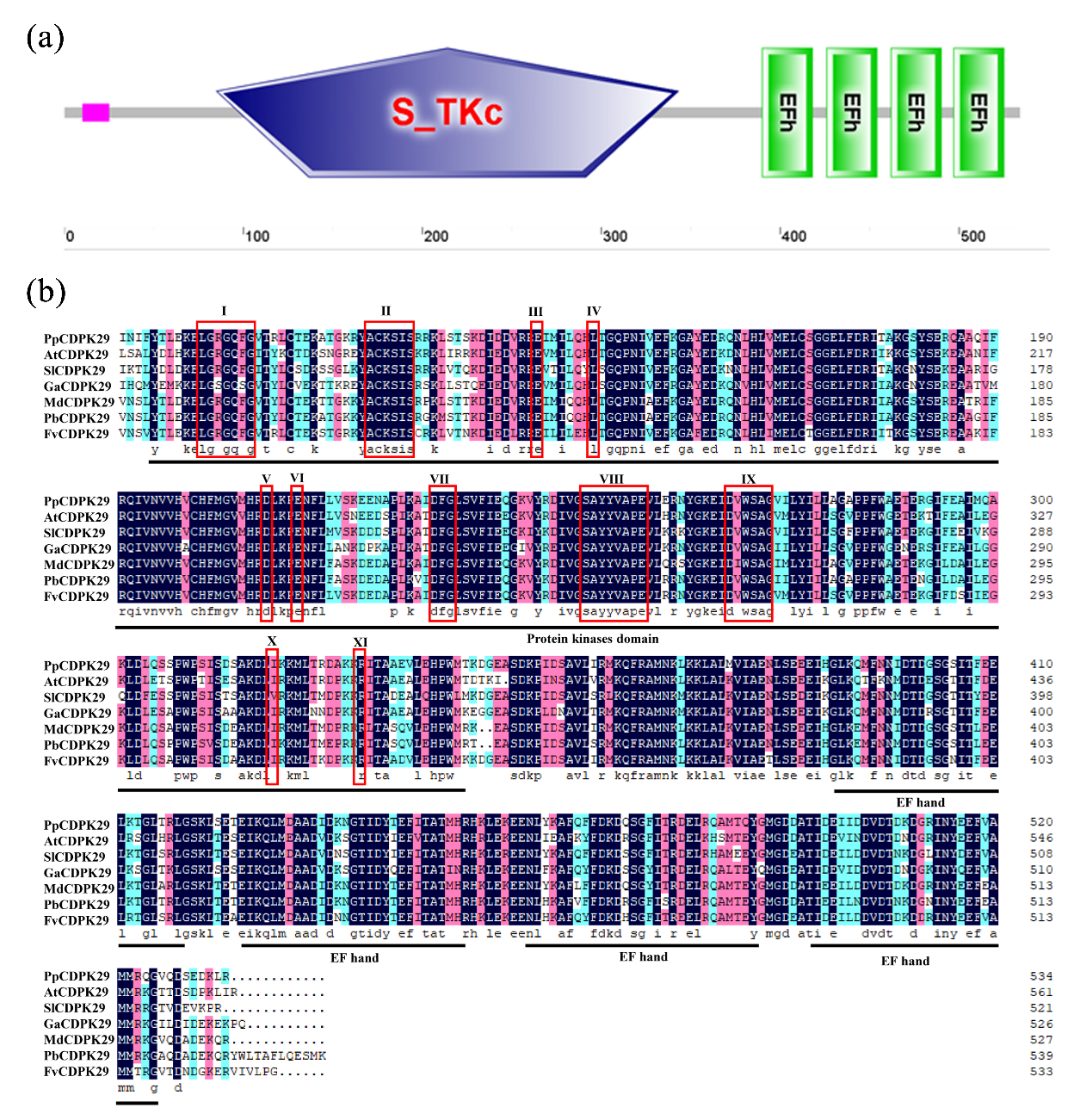


**Supplemental Figure S2** Characterization of PpCDPK29. (a) Protein structural characteristics of PpCDPK29. (b) Multiple alignments of amino acid sequence of PpCDPK29.


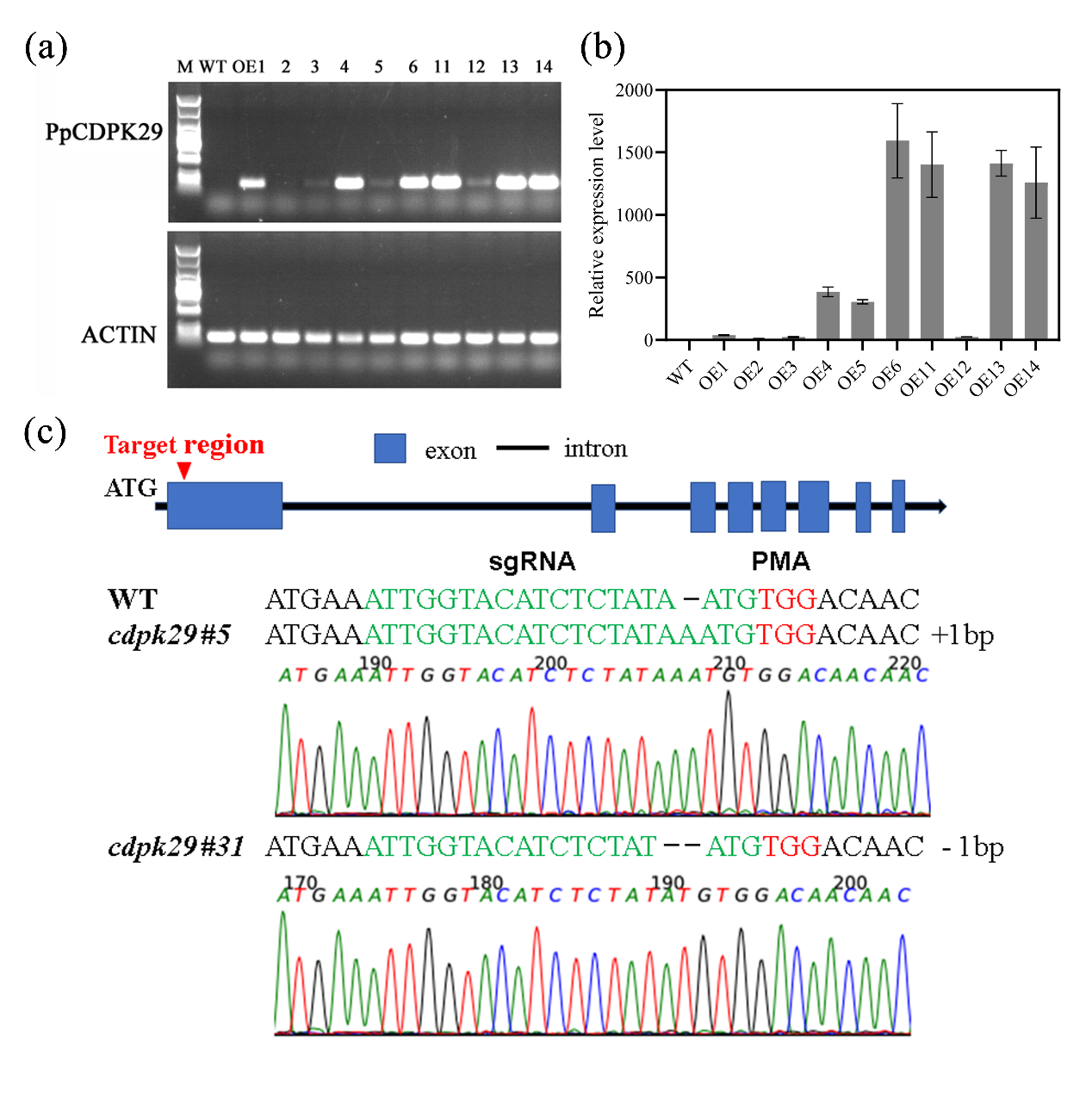


**Supplemental Figure S3** Identification of transgenic tomato positive seedlings. (a) The expression of *PpCDPK29* in positive plants of T1 generation was detected by semi-quantitative PCR. M: DNA marker. (b) Relative gene expression of *PpCDPK29* in tomato T1-generation positive seedlings. (c) Sequencing results of CRISPR/Cas9 tomato mutation lines.


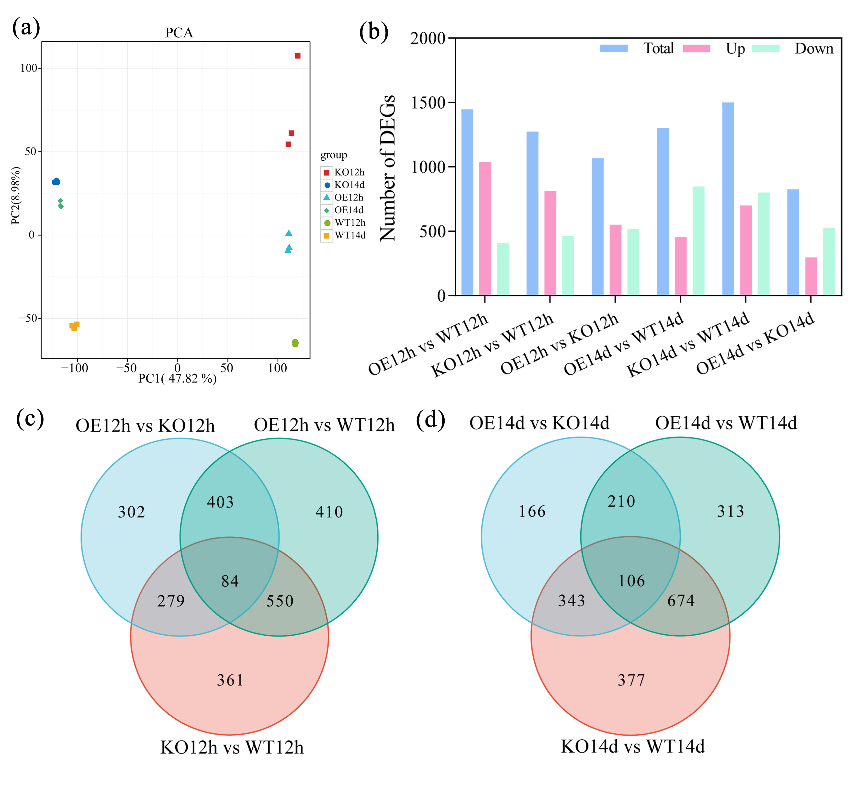


**Supplemental Figure S4** Analysis of DEGs between RNA-Seq groups. (a) PCA of RNA-Seq samples. (b) DEGs distribution histogram. (c) 12 h and (d) 14 d Venn diagrams of DEGs.


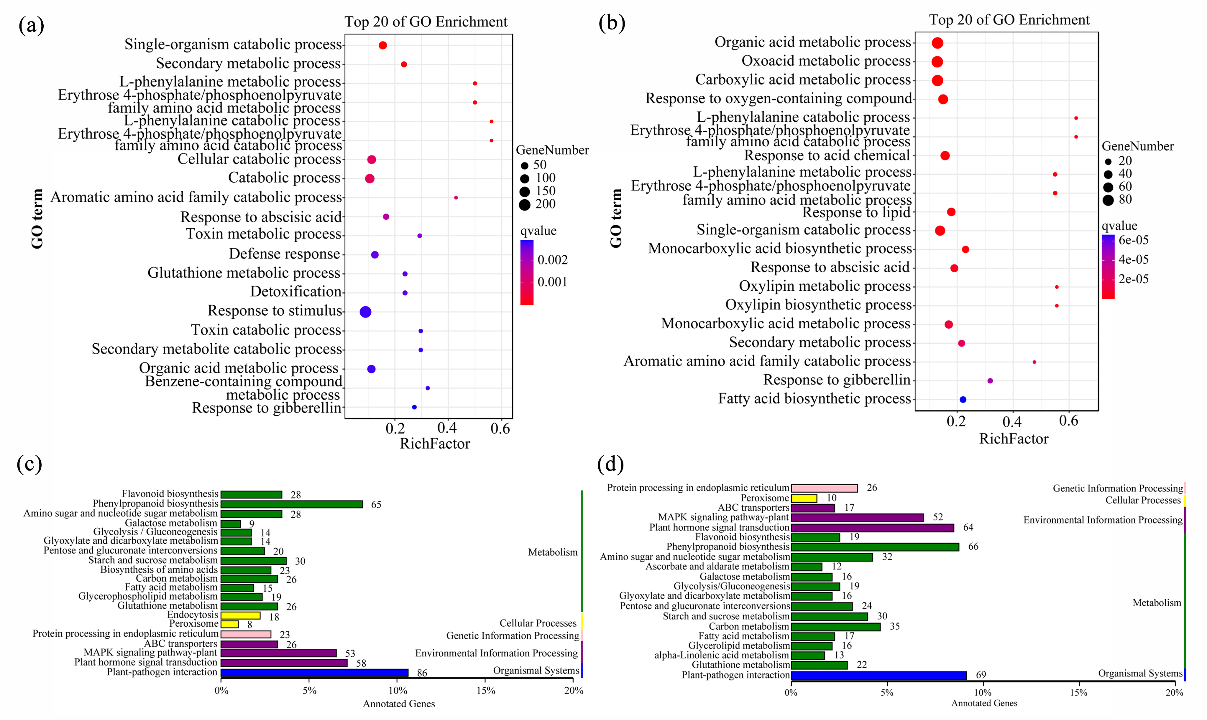


**Supplemental Figure S5** GO and KEGG analysis of DEGs in WT and transgenic tomato fruits. (a) 12 h and (b) 14 d enrichment analysis of DEGs in the ‘biological process’ GO subcategories. (c) and (d) KEGG analysis of DEGs in WT and transgenic tomato fruits.


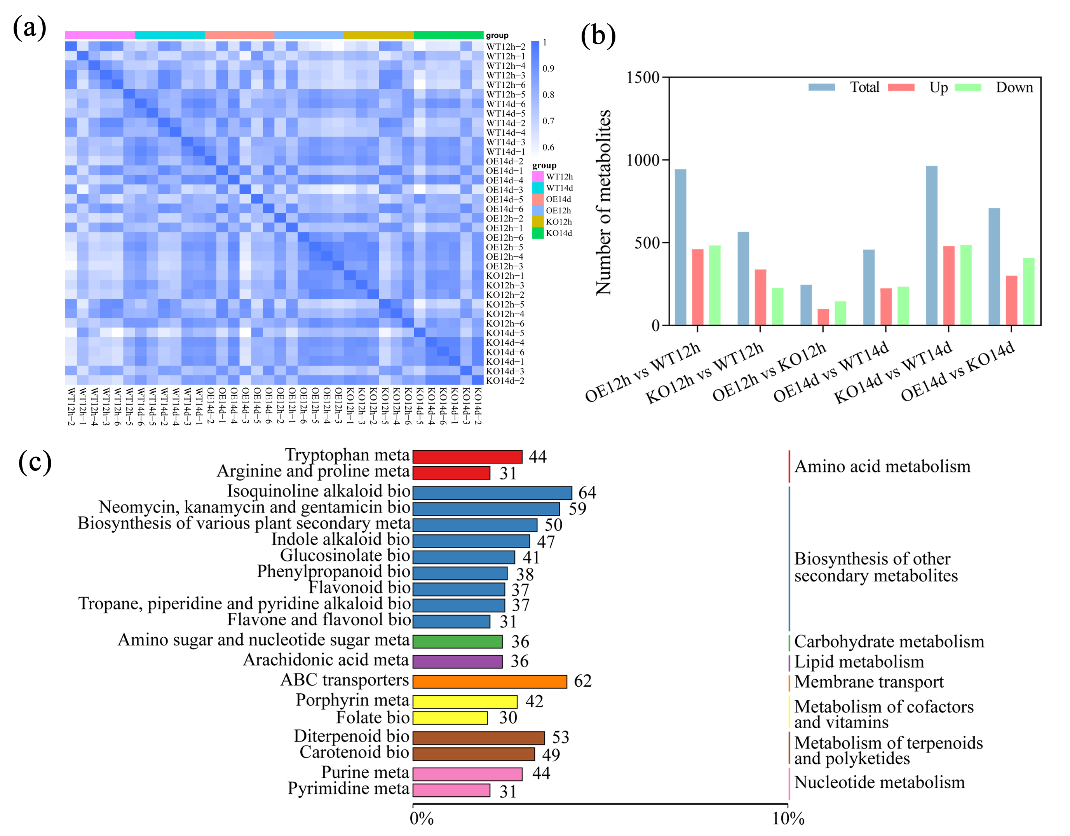


**Supplemental Figure S6** Metabolome differential metabolite analysis of WT and transgenic tomato fruits. (a) Correlation coefficient of each sample. (b) DEMs distribution histogram. (c) KEGG analysis of DEMs.


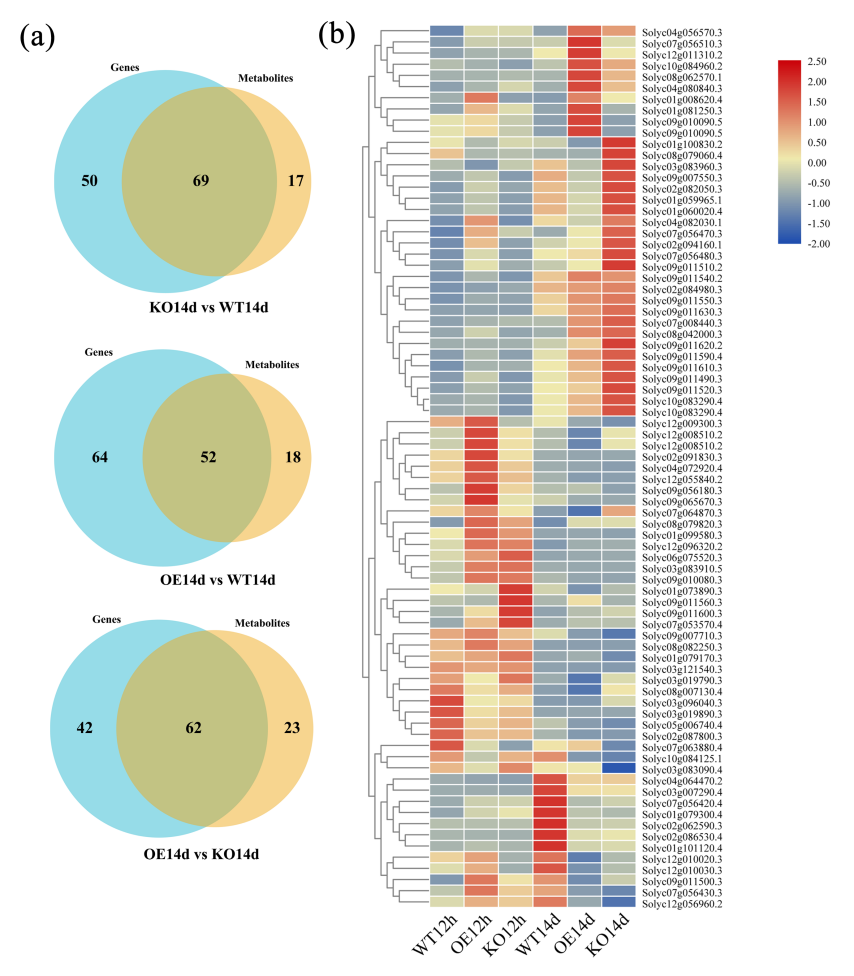


**Supplemental Figure S7** Cross-comparisons between transcriptomic and metabolome. (a) Venn diagram of DEGs and DEMs pathways. (b) Heat map of DEGs in Glutathione metabolism, Starch and sucrose metabolism and Galactose metabolism pathway.


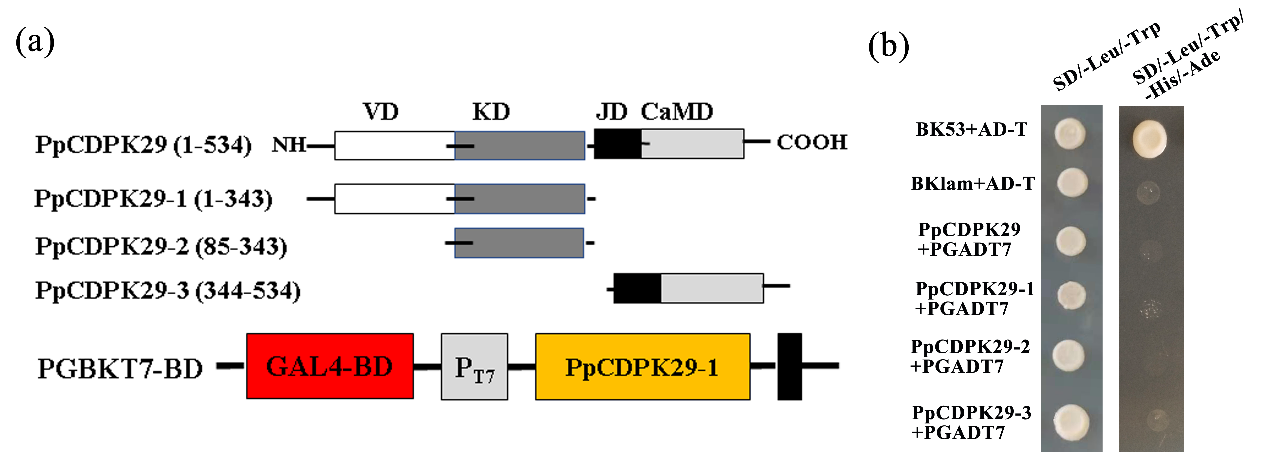


**Supplemental Figure S8** PpCDPK29 yeast two-hybrid vector construction. (a) Yeast two-hybrid protein fragment of PpCDPK29. (b) Validation of yeast self-activation of PpCDPK29.


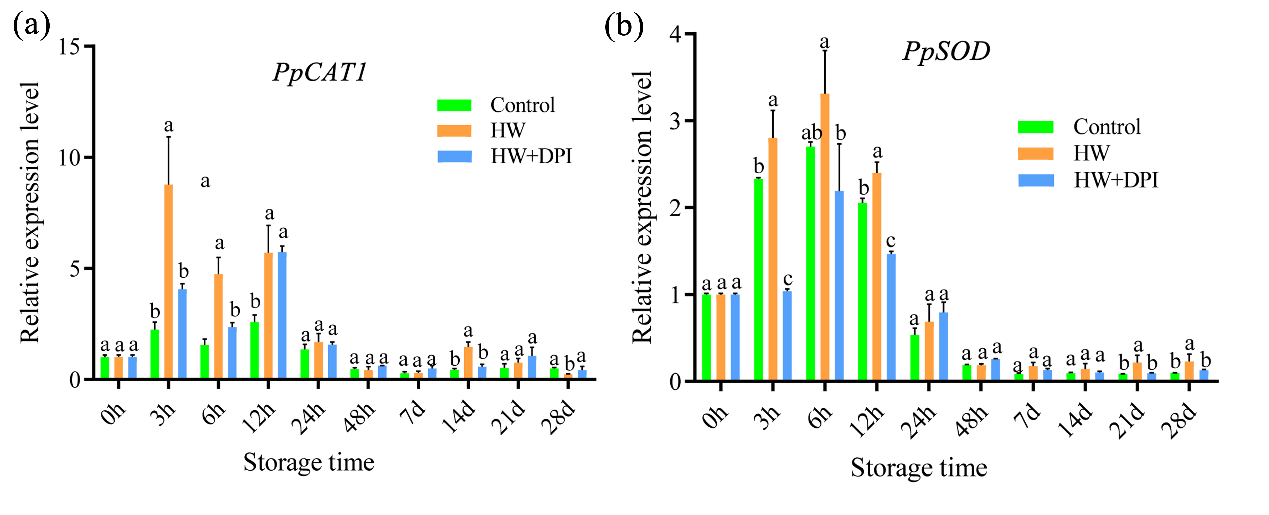


**Spplemental Figure S9** Gene expression analysis of *PpCAT1* (a) and *PpSOD* (b).


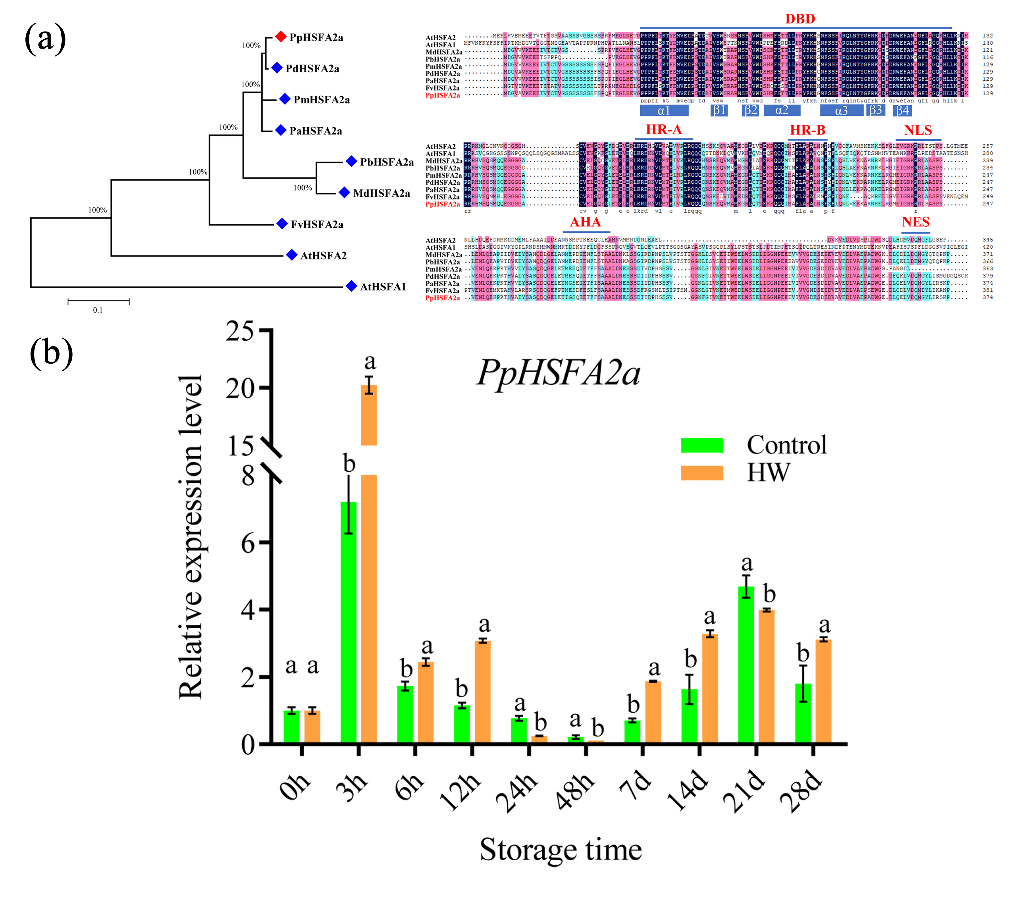


**Supplemental Figure S10** Identification and protein characterization of PpHSFA2a. (a) Phylogeny and amino acid multiple sequence analysis of the HSFA2 in peach and other species. (b) The relative expression of *PpHSFA2a* under HW treatment during cold storage.


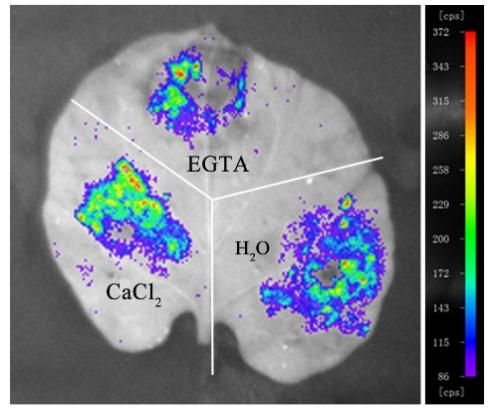


**Supplemental Figure S11** Effects of CaCl_2_ and EGTA treatment on the interaction between PpCDPK29 and PpHSFA2a.


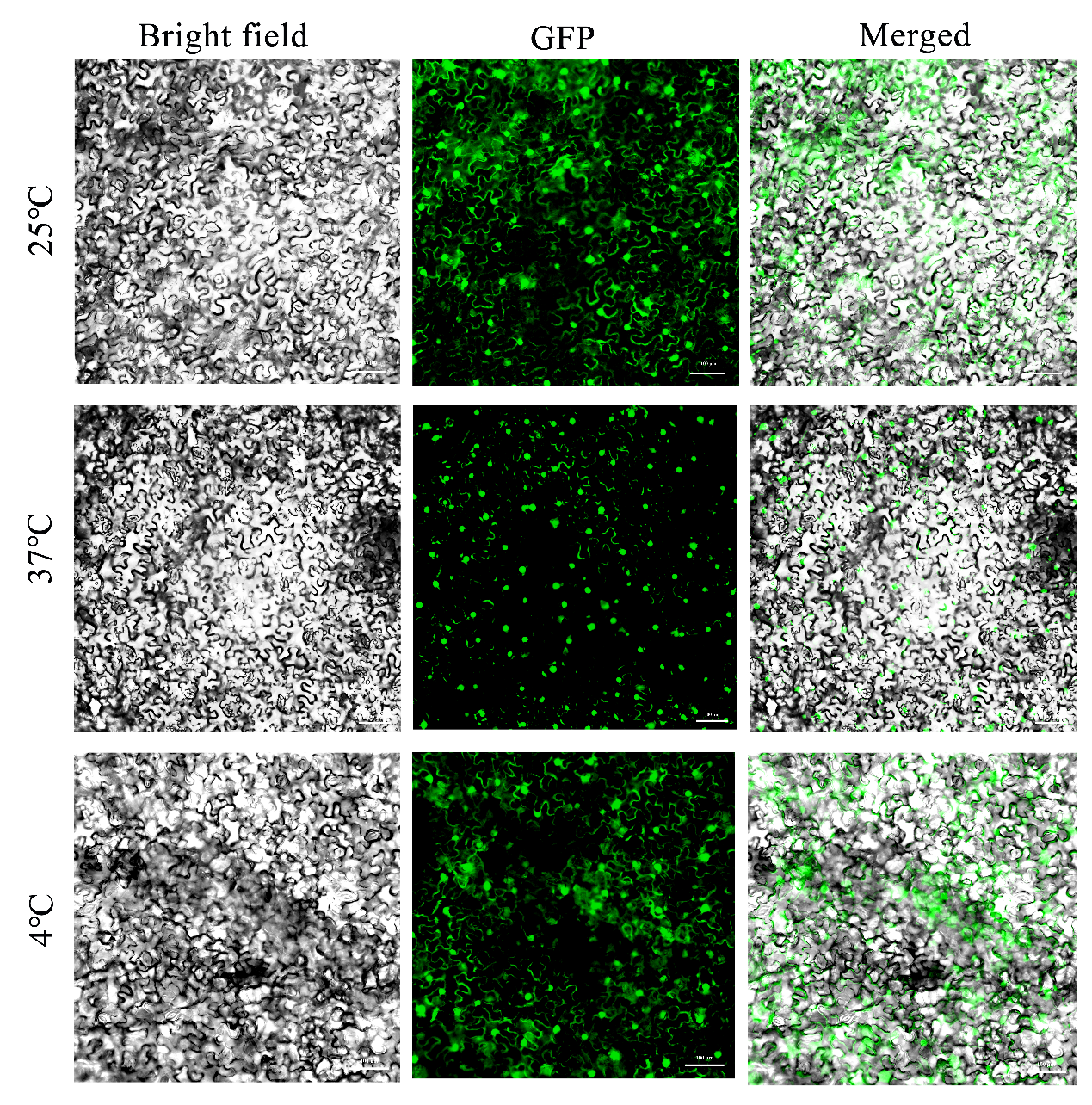


**Supplemental Figure S12** Effect of temperature on subcellular localization of PpHSFA2a (Bars: 100 μm). At normal room temperature, PpHSFA2a was located in the cytoplasm and nucleus, but after being treated at 37℃ for 4 h, PpHSFA2a was more transferred to the nucleus, and most of it was localized in the nucleus. However, after 4 h of low temperature treatment at 4°C, the localization change was not obvious, compared with normal temperature, only a small part was transferred to the nucleus.


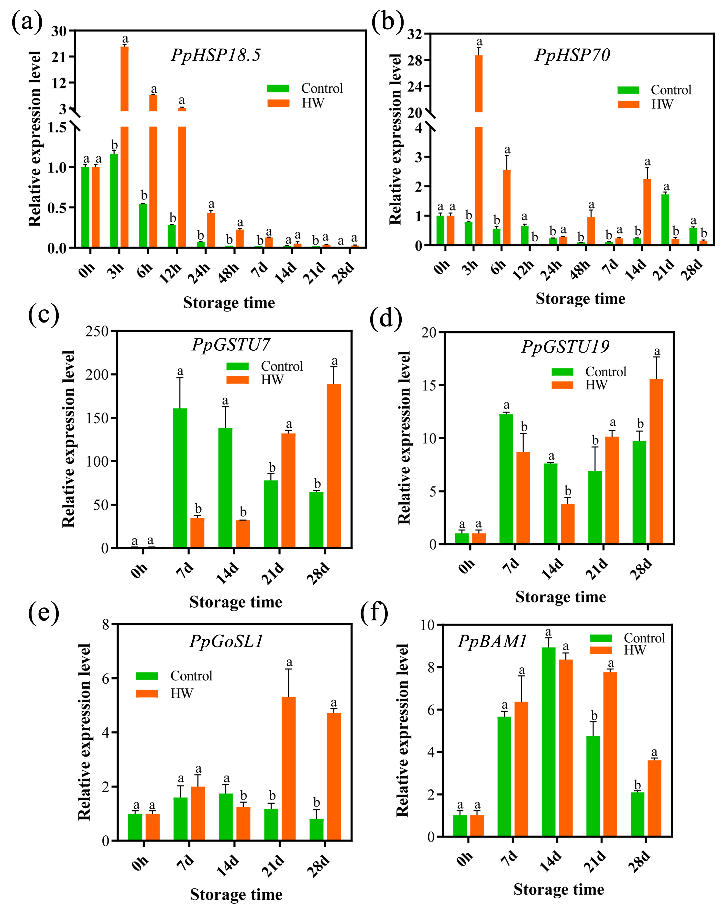


**Supplemental Figure S13** Expression level analysis of PpHSFA2a target gene. (a-f) The relative expression levels of *PpHSP18.5* (a), *PpHSP70* (b), *PpGSTU7* (c), *PpGSTU19* (d), *PpGolS1* (e), and *PpBAM1* (f).


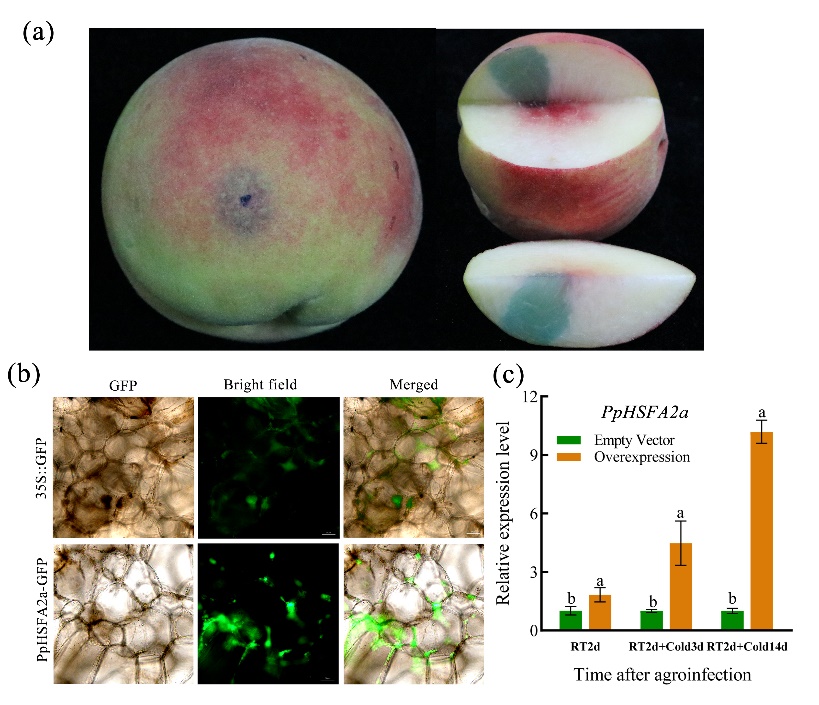


**Supplemental Figure S14** Transient overexpression and silencing analysis of *PpHSFA2a.* (a) The schematic diagram of *Agrobacterium* infiltration of peach fruits. (b) Fluorescence expression of GFP in peach fruit under transient infection. (c) The expression levels of *PpHSFA2a* after overexpression *PpHSFA2a.*


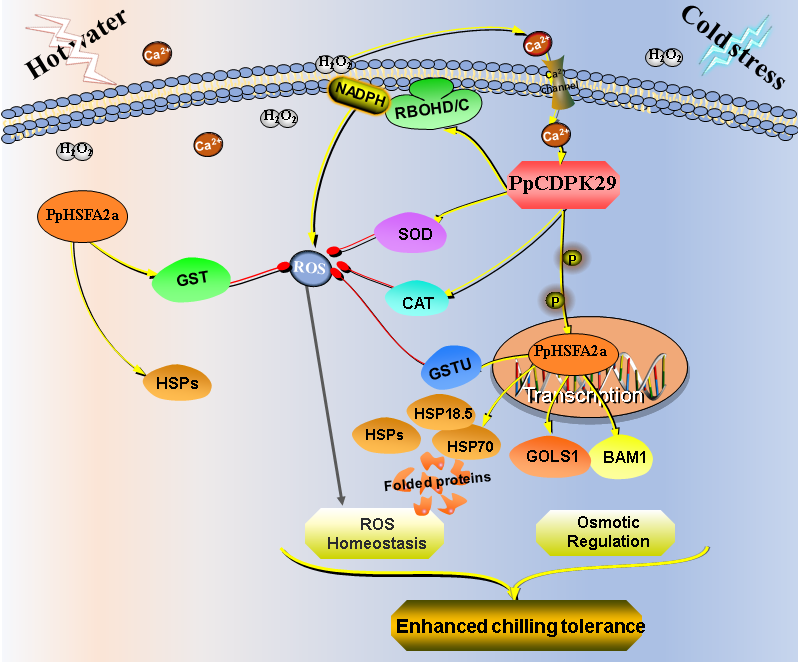


**Supplemental Figure S15** Proposed model for the PpCDPK29 signaling cascade involved in peach fruit chilling injury. HW treatment activates PpCDPK29. PpCDPK29 interacts with RBOHC/D to activate ROS, and interacts with SOD and CAT to scavenge excess ROS, thus maintaining ROS homeostatic balance. Meanwhile, PpCDPK29 can phosphorylate PpHSFA2a, and PpHSFA2a can directly regulate downstream defense-related genes alleviating chilling injury.

Supporting experimental procedures

Supplemental Method S1. **Measurement of chilling injury related indicators**

Internal browning was usually used to evaluate the chilling injury (CI) index. The browning area of the flesh was divided into five levels: 0 had no browning, 1 had 0-25% browning area, 2 had 25-50% browning area, 3 had 50-75% browning area, 4 had 75-100% browning area. The CI index was calculated by ∑ [(CI scale) × (number of fruit at that CI scale)] / (4 × total number of fruit).

Ion leakage and MDA contents were measured according to the method described in our previous study (Zhao et al., 2023). Eighteen flesh discs (2 mm thick and 6 mm in diameter) were collected from 6 fresh fruits and immersed in distilled water to record the initial conductivity of L0, then soaked for 30 min and recorded as L1, finally, the boiling water was boiled for 15 minutes and L2 was measured after cooling. The ion leakage was calculated as [(L1-L0) / (L2-L0)] × 100. The content of MDA was determined by the thiobarbituric acid method, and the content was presented as μmol kg^−1^ on fresh weight. The content of H_2_O_2_ was determined by titanium tetrachloride method. The absorbance was measured at 412 nm, and the results were expressed as μmol g^-1^ FW. O_2_^.-^ production rate was calculated from the sodium nitrite standard curve in μmol min^-1^ kg^-1^.

Supplemental Method S2. **SOD, CAT and APX activities measurement**

The activities of SOD, CAT and APX were measured according to described previously (Zhao et al., 2023). The 2 g peach flesh samples were fully ground in the pre-cooled extraction buffer and centrifuged at low temperature to prepare the supernatant. One unit of SOD activity was the amount of enzyme required to inhibit NBT reduction by 50% per minute per gram of sample. The change of 0.01 absorbance per gram of sample per minute at 240 nm was defined as 1 unit of CAT activity. The change of 0.01 in absorbance of the reaction solution at 290 nm per minute was defined as APX activity unit U. SOD, CAT, and APX activities were expressed as U kg^-1^.

Supplemental Method S3. **Generation of transgenic plants**

The p35S::PPCDPK29-GFP was used to generate *PpCDPK29* overexpression (OE) plants. The knockout line of the *SlCDPK29* in tomato was constructed using CRISPR/Cas9. The target sequence (ATTGGTACATCTCTATAATG) was designed on the first exon of *SlCDPK29* and built into the BG fast cas9 vector. Tomato genetic transformation was carried out using the Agrobacterium-infected leaf disc method, using Micro-Tom tomatoes as the transformation material. Tomatoes were grown in the plant incubator in controlled conditions (18 h light/6 h dark cycles, 25 ℃ day/20 ℃ night). The positive tomato seedlings overexpressing *PpCDPK29* were screened by antibiotic hygromycin, and high-expressing lines were screened by semi-quantitative PCR and qRT-PCR. The seedlings of T2 and T3 generations were screened by antibiotic hygromycin for subsequent experimental studies. Primers were designed near the sgRNA target site to identify SlCDPK29 mutants and the target editing pattern was analyzed by sequencing to obtain the homozygous CDPK29 mutant lines for subsequent experiments.

Supplemental Method S4. **DAB and NBT staining**

DAB staining was used to detect the accumulation of H_2_O_2_ in cells. The leaves were immersed in 1 mg/mL DAB for staining at room temperature for 8 h, decolorized with alcohol for 24 h, then rinsed with water and photographed for observation. NBT histochemical staining was used to detect the accumulation of O_2_^.-^ in cells. Leaves were soaked in 1 mg/mL NBT (PH 7.8 PBS buffer), stained at room temperature for 2 h, decolored with alcohol for 24 h, and finally washed with water.

Supplemental Method S5. **Transient expression in peach fruit**

Peach fruit transient expression was expressed using Agrobacterium-mediated transient transformation. *Agrobacterium* suspensions comprising p35S-PpHSFA2a-GFP fusion protein were slowly injected into the central on both sides of the peach fruit with a sterile syringe. After injection, peaches were put at 25 ℃ for 2 days, and then at low temperatures for 3 or 14 days. Peach fruit transient silencing was performed using Virus-induced gene silencing (VIGS) technology. The SGN VIGS tool (https://vigs.solgenomics.net/) was used to predict the specific CDS fragment of *PpHSFA2a*. The 300 bp CDS of PpHSFA2a was connected to the pTRV2 vector. Next, the *Agrobacterium* of pTRV1 and PpHSFA2a-TRV2 were mixed in a ratio of 1:1 to infect the peach fruit, and then stored at 4 ℃ for 14 days after incubation at 25 ℃ for 2 days. Fruit samples near the injection site were taken for physiological indicators and gene expression determinations.
